# Equine Parvovirus-Hepatitis Population Dynamics in a Single Horse over 16 Years

**DOI:** 10.3390/v17070947

**Published:** 2025-07-04

**Authors:** Alexandra J. Scupham

**Affiliations:** Animal and Plant Health Inspection Service, Center for Veterinary Biologics, Ames, IA 50010, USA; alexandra.scupham@usda.gov; Tel.: +1-515-337-6441

**Keywords:** Theiler’s disease, equine parvovirus-hepatitis, ecology, evolution

## Abstract

Many viruses mutate rapidly to adapt to host defenses, and for some of these viruses, the result is long-term infection in individual hosts. The work described here examines the infection and long-term maintenance of a newly identified virus, equine parvovirus-hepatitis (EqPV-H), in an individual horse. This description is possible because of a hypervariable region in the capsid gene; sequence variants were tracked by high-throughput sequencing of serum samples taken over a 16-year period. The data support the hypothesis that EqPV-H infection resulted in a sequence variant bottleneck. The continuing infection evolved into a complex viral population showing a pattern of emergence, dominance, and recession with replacement. This is the first temporal description of the capsid gene evolution of EqPV-H in a single animal.

## 1. Introduction

Theiler’s disease (TD), a hepatitis of horses associated with the administration of tetanus antitoxin and other equine serum products, was first described over one hundred years ago [1]. TD causes a wide range of clinical signs, from subclinical through acute hepatitis resulting in death within days of onset. Numerous viruses have historically been proposed as the causative agent, but none have shown both liver tropism and disease correlation [2]. In 2018 a novel parvovirus was detected by high-throughput sequencing (HTS) of the serum of an afflicted horse as well as from the vial of tetanus antitoxin that had been administered to the patient prior to the onset of clinical signs [3]. Accumulating evidence suggests that the new virus, named equine parvovirus-hepatitis (EqPV-H), is the etiologic agent [4]. This evidence includes the robust correlation of the virus with TD symptoms and the presence of the virus in hepatic lesions [5,6,7,8].

Parvovirus capsids are formed from 60 copies of 2–3 splice variants of coat protein VP, depending on the family [9]. The variants consist of the full-length protein VP1 and N-truncated versions thereof. VP2, the largest of the N-truncated variants, constitutes the major structural protein of many parvovirus capsids. The well-studied human parvovirus B19 generates capsids with 5% VP1 and 95% VP2. The parvovirus VP1-specific N-terminus (VP1u) contains phospholipase A2 (PLA2) and calcium-binding domains which are thought to be packaged inside the capsid, emerging through the fivefold-axis pore when an infecting virus encounters the host cell’s acidic endosome [10,11]. These domains are essential for escape from the endosome, and in human parvovirus B19 and canine parvovirus (CPV), they are targets for neutralizing antibodies [12].

RNA (and some ssDNA) viruses with extremely high rates of mutation are termed quasispecies (QS) [13]. QS mutation rates range from 10^−5^ to 10^−3^ mutations per nucleotide (nt) per infectious cycle, causing them to exist as a population of closely related sequences within a host [14,15]. Mutation rates vary across a given viral genome depending on locus roles in the biology of the virus—for example, the hepatitis C virus 5′ UTR essential for viral replication is relatively conserved, whereas the hypervariable region 1 of the E2 gene provides a mode of host immune escape [16,17,18,19]. Since Epstein and Eigen first posited the existence of QS, a suite of classical ecology tools has been adapted for use with molecular data for the examination of QS biology, ecology, and epidemiology [20,21].

With few exceptions, investigations into EqPV-H have not addressed genetic variability in the VP gene [22,23,24,25]. The genomic mutation rates of various parvoviruses were previously estimated to be 1–2 × 10^−4^ mutations/nt/year [26,27]. The genomic mutation rate of EqPV-H has not yet been explored. However, a hypervariable (HV) region is located between the VP1u PLA2 locus and the VP2 sequence encoding the glycine-rich pore (Supplementary Sections S1 and S2). The mutation rate at this locus is estimated to be 4.6 × 10^−3^ mutations/nt/year; therefore, the current work investigates the biology and ecology of this new virus using ecological tools developed for studying QS [28,29].

In this study, high-throughput sequencing (HTS) was used to examine serum samples drawn from a single horse over a period of 16 years. Long-term carrier status was confirmed, and the sequence variability showed that the introduction of the virus to this horse shows evidence of a genetic bottleneck, similar to those found in studies of hepatitis C virus (HCV) and human immunodeficiency virus (HIV) [30,31,32]. An evolving community with viral load and composition changes over time then developed.

## 2. Materials and Methods

### 2.1. Samples

The Center for Veterinary Biologics (CVB) obtained ten serum samples donated by the owner of an individual healthy horse (horse A). Sera had been taken over the course of 16 years and stored frozen at −20 °C. Upon transfer to CVB, the samples were thawed, divided into 1 mL aliquots, and stored at −20 °C.

### 2.2. Luminescent Immunoprecipitation System (LIPS)

The reporter antigen for a LIPS antibody assay was generated using the TNT SP6 Quick-Coupled Transcription/Translation System (Promega, Madison, WI, USA) with a protocol adapted from Lahner 2017 [33,34]. First, the VP2 region of the VP gene was amplified from DNA of sample 2004 using primers VP2EcoKoz 5′-TTTGCAGAATTCGCCATGGACACGTCGCTGCATTCTGA-3′ and VP2XbaPolyAR 5′-GTCGACTCTAGATTTTTTTTTTTTTTTTTTTTTTTTTTTTTTCTGTAACTTAGAAGGAACTG-3′. This amplicon was cloned into vector pNLF1-N (Promega) *EcoR*I and *Xba*I sites. Purified plasmid from a clone containing the correct insert was used for in vitro generation of nanoluciferase-labeled EqPV-H VP2 protein (Nluc-VP2) using the TNT T7 Quick Master Mix per the manufacturer’s instructions (Promega).

Light units (LU) associated with serum anti-EqPV-H Ab were measured using the Nano-Glo Dual-Luciferase Reporter Assay System (Promega). The Nluc-VP2 chimeric protein concentration was adjusted to 1.3 × 10^5^ LU/μL, and the LIPS assay was performed as described previously [35]. Briefly, 6 μL of equine serum was combined with 75 μL Nluc-VP2 and 18 μL PBST, and then 33 μL of the mixture was dispensed into triplicate wells of a pre-wetted MultiScreenHTS Filter Plate (Sigma-Aldrich, St. Louis, MO, USA). The samples were incubated at room temperature for 2 h in the dark, and then 50 μL of 5% glycine-blocked rProtein A Sepharose (PAS) (Cytiva, Marlborough, MA, USA) was added to each well. An additional hour of 14 °C incubation was performed at 300 rpm in the dark. The wells were washed 12 times with 150 μL PBST using a manifold, and then the plates were centrifuged at 500 rpm for 5 min to remove excess liquid. Light was generated by adding 65 μL per well of Promega Nanoluc luciferase substrate diluted 1:325. LU was measured using a Spectramax M5 with Softmax v7 (Molecular Devices, San Jose, CA, USA). Previously described EqPV-H-naïve and -positive equine sera ES73 and ES5 were used as controls on every plate [36]. LU measurements from triplicate reactions were averaged, and standardized units were calculated as

((Sample Avg-Avg Neg Control)/(Avg Pos Control-Avg Neg Control))100

### 2.3. Viral Nucleic Acid Detection

Nucleic acid was extracted from 200 μL serum aliquots using a Promega Maxwell RSC robot with the RSC Viral Total Nucleic Acid Purification Kit. DNA was eluted in 60 μL of nuclease-free water.

To quantify EqPV-H genomes in the serum samples, digital PCR (dPCR) was performed as described previously using real-time primers and probe [5,36]. Reactions were cycled in a QIAcuity dPCR thermocycler using the QIAcuity Probe PCR Kit master mix (Qiagen, Hilden, Germany) [30]. Then, 40 μL reactions were added into 24-well 26K Nanoplates (Qiagen). Reactions contained 10 μL of template, 520 nM of each primer (EqPV-321F: ATGCAGATGCTTTCCGACC; EqPV-3386R: GCCCCAGAAACATATGGAAA), and 260 mM of HPLC-purified probe (EqPV-3310: 56-FAM/ACCGTAGCG/ZEN/GATTCGGGATCTGC/31ABkFQ). Cycling conditions included an initial 2 min, 95 °C denaturation followed by 40 cycles of 30 s at 95 °C and 1 min at 58 °C.

### 2.4. Sequencing and Data Preparation

A 2.1 kb partial VP gene (73.0% of the VP gene, equivalent to 35.6% of the genome) was amplified from each undiluted DNA extraction to provide template for high-throughput sequencing (HTS). Amplifications used Qiagen HotStarTaq Plus Master Mix with an initial denaturation of 94 °C for 5 min followed by 43 cycles of denaturing at 94 °C for 10 s, annealing at 56 °C for 20 s, and elongation at 72 °C for 3 min. A final 0.4 μM concentration of primers EqPV-HF10 5′-CCTGACCTGAAATGGATGAA-3′ and EqPV-HR4 5′-GGGATAGTGGGTTTTGTCAT-3′ and 5% *v*/*v* DNA completed the reactions. Three to six 33–50 μL reactions were performed for each sample, such that 30 μL of 30 ng/μL of each amplicon was available for HTS. After amplification, faint bands were visible on agarose gels. The number of reactions necessary to generate HTS template depended on the viral load in each sample. Amplicons were pooled and purified using a Qiagen QIAquick kit per the manufacturer’s instructions, and then submitted for Nextera tagmentation and Illumina miSeq HTS. This entire process was performed in duplicate for each sample.

Reads from the duplicate HTS data sets were merged for analysis. Reads were paired, trimmed with an error probability limit of 0.01, and filtered to allow a maximum of one low-quality base per sequence. The paired reads were then merged using the Geneious Prime BBMerge tool (v. 2023, Biomatters Ltd., Auckland, New Zealand) [37]. Geneious Mapper was used to map both merged and unmerged reads to either a conserved (CS) 126 base-pair (bp) in-frame locus from the EqPV-H VP gene (accession number MG136722, nucleotides 2260–2385), or 126 bp of a hypervariable (HV) region (nt 928–1053). Sequences were masked to remove nt outside each 126 bp target, and then sequences < 126 bp long were removed. Reads were deduplicated and each unique sequence and the number of reads per unique sequence recorded (Table 1). Finally, the data sets were trimmed to remove sequences comprising ≤0.3% of each data set. These unique sequences, herein referred to as haplotypes, were then collected into FASTA format for analysis. Throughout the manuscript, the curated haplotypes as a group are termed the community or population.

The two 126 bp regions were chosen for analysis rather than the complete 2.1 kb amplicon because the preponderance of individual reads generated by Illumina miSeq technology is only 251 nt in length. The assembly of these fragments generates a mosaic of overlapping reads displaying the full complement of variability; therefore, polymorphisms observed at either end of the amplicon cannot be attributed to a given 2.1 kb haplotype.

### 2.5. Phylogenetic Analyses

Haplotypes were aligned using the MAFFT algorithm in Geneious Prime, and then dendrograms were generated with Randomized Axelerated Maximum Likelihood (RaxML) using 300 bootstrap replications [38,39]. Dendrograms were divided into clades discriminated at a sequence similarity of 4 nt. Unique identifiers were assigned to each haplotype based on the clade and order of emergence. Haplotypes from the 126 bp CS and 126 bp HV region were analyzed separately.

### 2.6. Statistical Analyses

Population diversity indices are composed of measurements of haplotype richness (S, the number of distinct haplotypes in a sample) and relative abundance (p^i^). For the Gini–Simpson Index of diversity (GSI) on a scale of 0 to 1, low values indicate a low probability that two haplotypes selected at random will be different. The measurement of evenness (J) on a scale of 0–1 compares the ratios of all haplotypes in a sample. Calculations of evenness were performed by Pielou’s evenness index [40]. Greater evenness is indicated by higher values.

Mutation frequency was calculated for the 2011–2019 data as the number of haplotypes divided by the number of nucleotides in the alignment, per year [41].

Frequency charts of the haplotypes in each sample were generated using the clades identified in the dendrograms [42]. Within a clade, haplotypes were plotted by the year of emergence and then in order of decreasing frequency.

The Mixed Effects Model of Evolution (MEME) and Fast Unconstrained Bayesian AppRoximation (FUBAR) tools were used to measure episodic selection pressure on both the CS- and HV-region amino acids [43,44]. These tools can be found on the Datamonkey Adaptive Evolution Server (https://www.datamonkey.org/, accessed on 1 January 2024). The methods compare nonsynonymous (dN) and synonymous (dS) rates of change using either a mixed-effects maximum likelihood approach or a Bayesian approach, respectively. Analyses were performed on the MAFFT alignments.

The index of commons (C*_m_*) and overlap index (O*_v_*) between samples were calculated as described previously [45]. C*_m_* is a measure of the fraction of haplotypes common to QS in two different samples, and O*_v_* is the sum of the minimum proportion of these common haplotypes.

## 3. Results

### 3.1. Target Region Identification

Conserved (CS) and hypervariable (HV) regions of the EqPV-H VP gene were identified using an alignment of 16 EqPV-H sequences retrieved from GenBank on 10 February 2023 (Section S1). The CS locus is composed of nucleotides 2260–2385 (amino acids 754–795) with reference to GenBank accession MG136722. The HV region encompasses nucleotides 928–1054 correlating to amino acids 310–351.

### 3.2. Viral Load and Antibody Response

**Figure 1:**
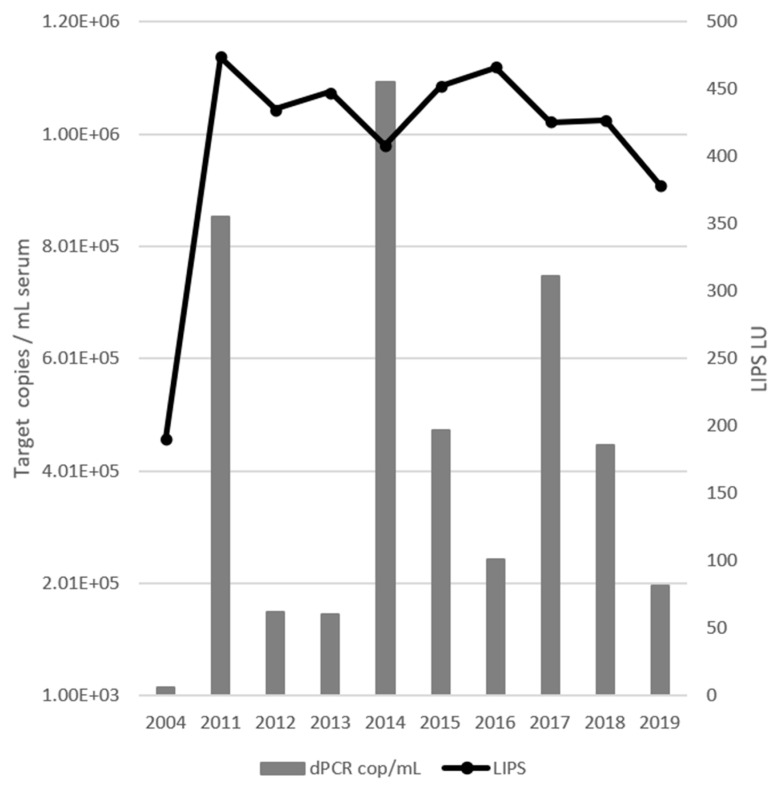
Digital PCR (dPCR) EqPV-H quantification (bars) compared to the LIPS antibody titer (line). The initial sample has lower values for both measurements than samples taken at any other time. Periodicity of viral load is visible as three peaks in 2011, 2014, and 2017.

Digital PCR analysis determined that horse A had a 16-year continuous EqPV-H infection (Figure 1). The viral load was initially 1.64 × 10^4^ genomes/mL serum, but thereafter fluctuated with a three-year periodicity, ranging from 1.46 × 10^5^ to 1.09 × 10^6^ genomes/mL serum. The periodicity presented as a spike in genomes/mL in one year followed by two years of a >1.5-fold decrease in viral load. The antibody response to EqPV-H infection showed a low titer of 190 LIPS LU in 2004, followed thereafter by a sustained response averaging 435 LU (Figure 1).

### 3.3. HTS Analysis

HTS data analyses were performed for each sample at both the CS and HV loci (Table 1 and Table 2). Between 3 and 5.5 million HTS reads were generated for each sample. Each HTS data set had a confidence mean ≥38.3, indicating high-quality runs.

An average of 50.5 thousand reads per sample (~1% of the total number of reads generated) mapped to each of the CS and HV loci. In the combined ten-sample data set, 8056 distinct CS haplotypes were distributed with an average of 1496 haplotypes per sample (Table 1, Supplementary Section S3). Of these 8056 distinct CS haplotypes, 38 were present after the 0.3% prevalence trimming; i.e., each represented ≥0.3% of the total number of mapped reads. Within each sample, 7–12 of these dominant haplotypes were present. In comparison, the HV haplotype pool was composed of an average of 2891 unique sequences per sample allocated from 20,839 total haplotypes (Table 2, Supplementary Section S4). Of these dominant haplotypes, as few as 5–25 per sample were retained after the 0.3% trimming step. The dominant haplotypes comprised 82% of CS and 93% of the total number of HV reads in each data set. The reported haplotype frequencies are based on the untrimmed data set.

**Figure 2:**
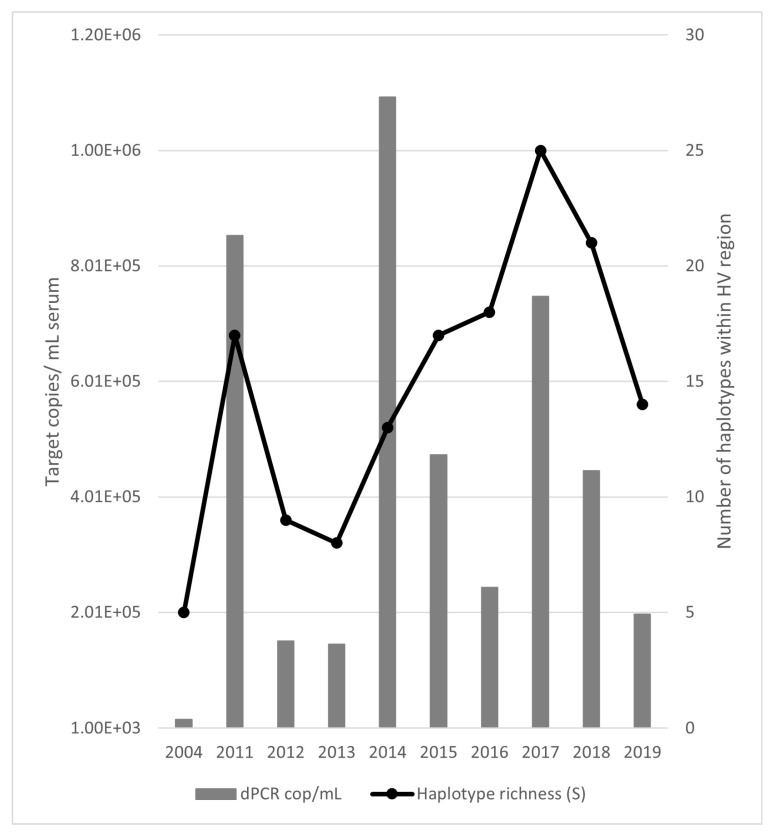
Digital PCR (dPCR) EqPV-H quantification (bars) compared to haplotype richness at the HV locus (line). The initial sample has lower values for both measurements than samples taken at any other time. Richness coincides with viral load in 2011 and 2017 but not 2014.

Figure 2 shows the juxtaposition of the HV dominant haplotype number against the calculated viral load from Figure 1. Although the viral load was highest in 2014, there was no corresponding peak in dominant haplotype numbers.

### 3.4. RAxML Trees

**Figure 3:**
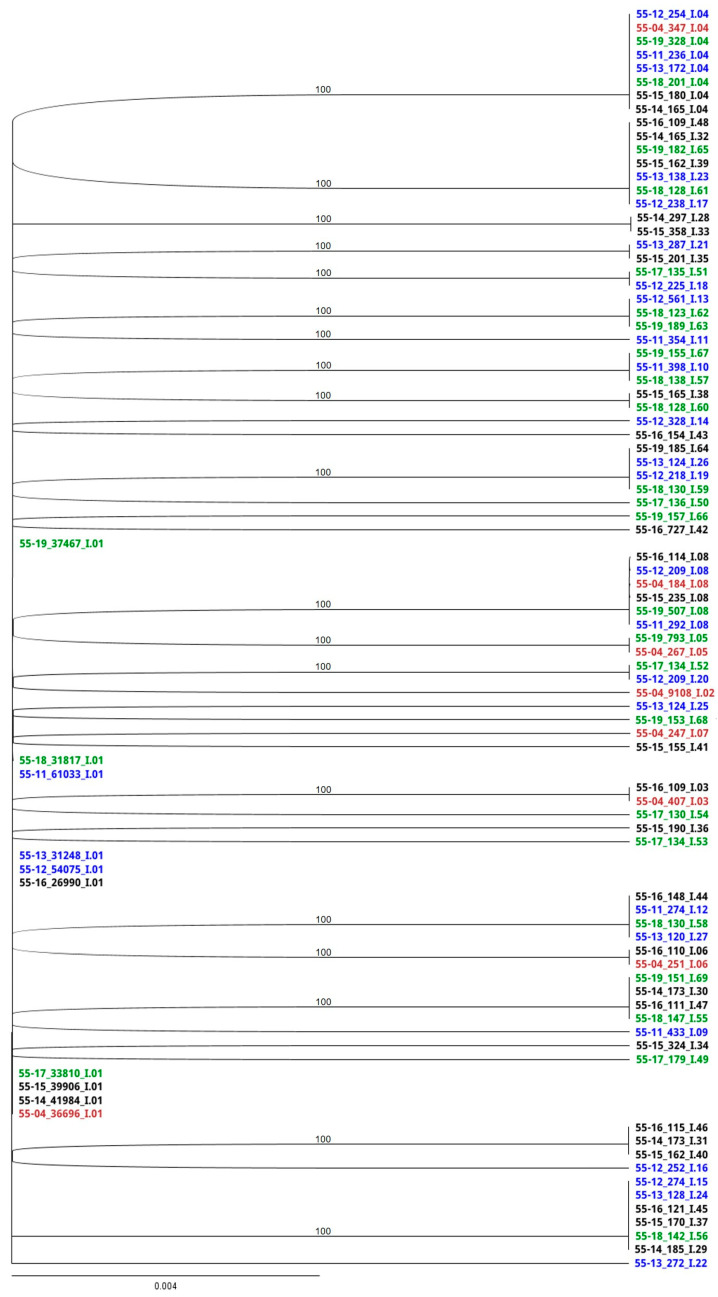
RaxML analysis of the conserved region. Sequences identified in 2004 are indicated in red. Haplotypes associated with years 2011–2013 are noted in blue, 2014–2016 in black, and 2017–2019 in green. Three-part haplotype designations are composed of fields separated by underscores. Designations begin with the horse identification number and two-digit year. Numbers in the second field represent the number of replicates of that haplotype found in the high-throughput sequencing data. The final field indicates the clade and order of emergence.

**Figure 4:**
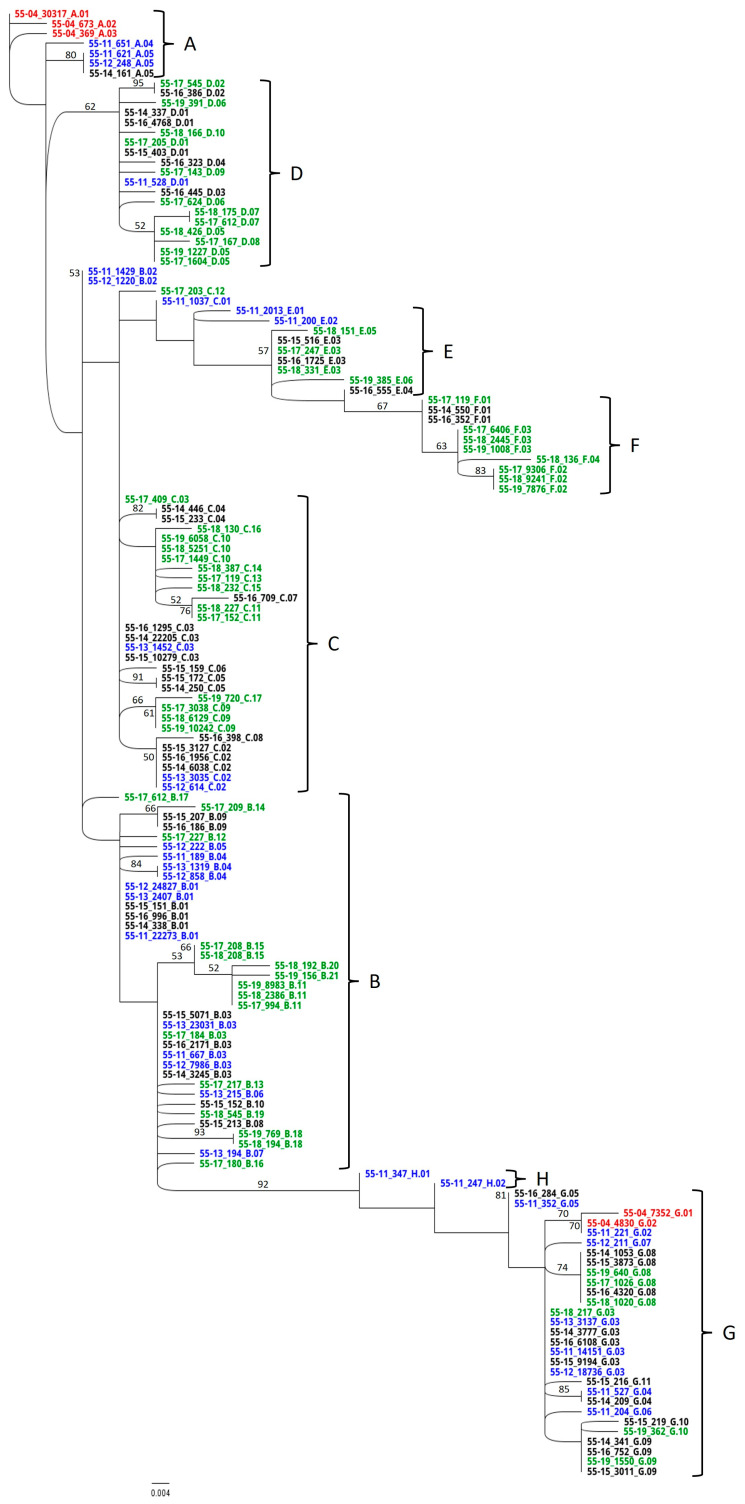
RaxML analysis of the hypervariable-region haplotypes. Sequences identified in 2004 are indicated in red. Haplotypes associated with years 2011–2013 are noted in blue, 2014–2016 in black, and 2017–2019 in green. Three-part haplotype designations are composed of fields separated by underscores. Designations begin with the horse identification number and two-digit year. Numbers in the second field represent the number of replicates of that haplotype found in the high-throughput sequencing data. The final field indicates the clade and order of emergence.

Phylogenetic trees depict the dominant members of the EqPVH community as identified by sequence analysis. Clades were discriminated at a 4 nt difference cutoff (Figure 3 and Figure 4). The scale bar length of 0.004 indicates the horizontal equivalent of a 0.5 nt difference. Haplotype names as shown in the RAxML trees are divided into three parts separated by underscores. The names start with the horse identification number and the two-digit year in which the sample was taken. The number in the second position indicates the number of copies of that sequence detected in the HTS data set from each sample. The final part of the name indicates the clade and order of emergence.

CS haplotypes formed a single clade regardless of sample year (Figure 3). The initial community (2004, red) contained eight different, closely related haplotypes, I.01 (36,696 reads) through I.08 (184 reads). Sequences I.02–I.08 were all a single nt different from I.01 and 2 nt different from one another. Four were a single-amino-acid different from I.01. Thereafter, I.01 continued to be the master haplotype while mutations accumulated randomly such that no sequence was more than 1 nt different from I.01. Thirty-eight total haplotypes were detected, some in multiple years.

Phylogenetic analysis of the HV haplotypes (Figure 4) identified eight separate clades, A–H. Two distinct groups of dominant sequences in the 2004 sample fell into clades A and G (red). In the intervening 7 years between the 2004 and 2011 samples, clade A generated daughter clades B-F while clade G generated the short-lived clade H. No 2004 haplotype except G.02 was detected in subsequent years. By 2015, no haplotypes from clade A were detected, while clades B, C, and G were represented every year from 2011 onward. In 2014, clade E appeared to spawn its own daughter, clade F.

### 3.5. Frequency Charts

**Figure 5:**
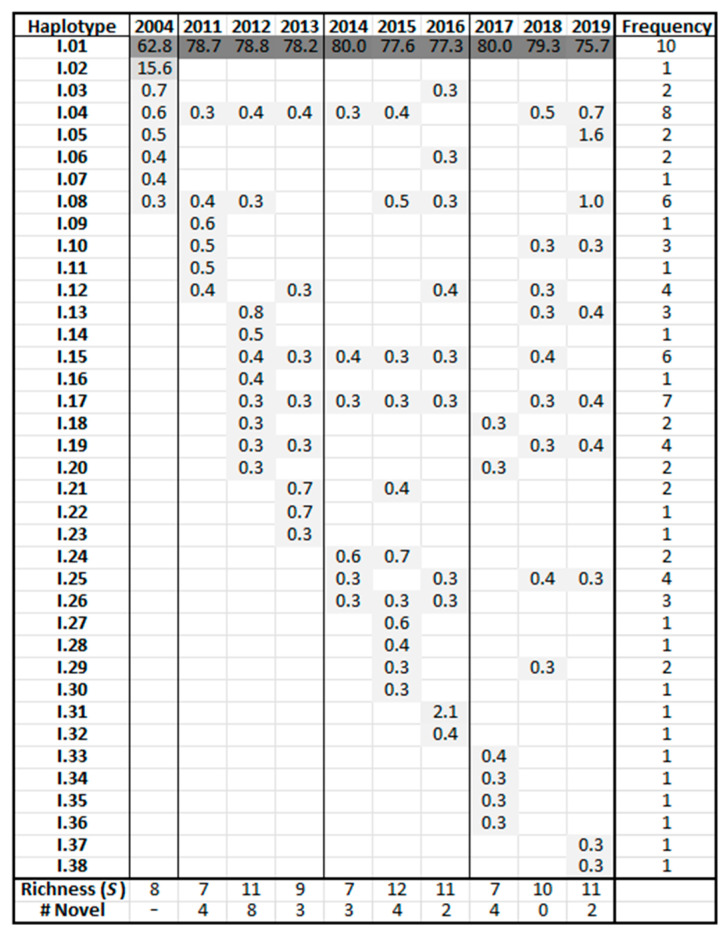
Frequency chart for conserved-region haplotypes. Haplotype names are truncated to the clade and order of emergence. Haplotypes are sorted by year and then in order of decreasing frequency. Values indicate the frequency of a given haplotype in each sample prior to the 0.3% prevalence trimming.

Haplotype frequency charts display abundance variations for individual haplotypes over time. Figure 5 shows the frequencies of the CS relative abundances where haplotypes are sorted by year of emergence and then in order of decreasing frequency. This information, while shown in the phylogenetic trees (Figure 3 and Figure 4), is more clearly represented in the frequency charts. All dominant haplotypes are represented. The initial 2004 sample contained two predominant CS-region haplotypes, I.01 (62.8%) and I.02 (15.6%). Thereafter, master haplotype I.01 predominated, with no other sequence gaining greater than a 2.1% foothold in the population. Population development of the non-master haplotypes was evident in the following years, with richness decreasing after every major upheaval correlating with the aforementioned periodicity.

**Figure 6:**
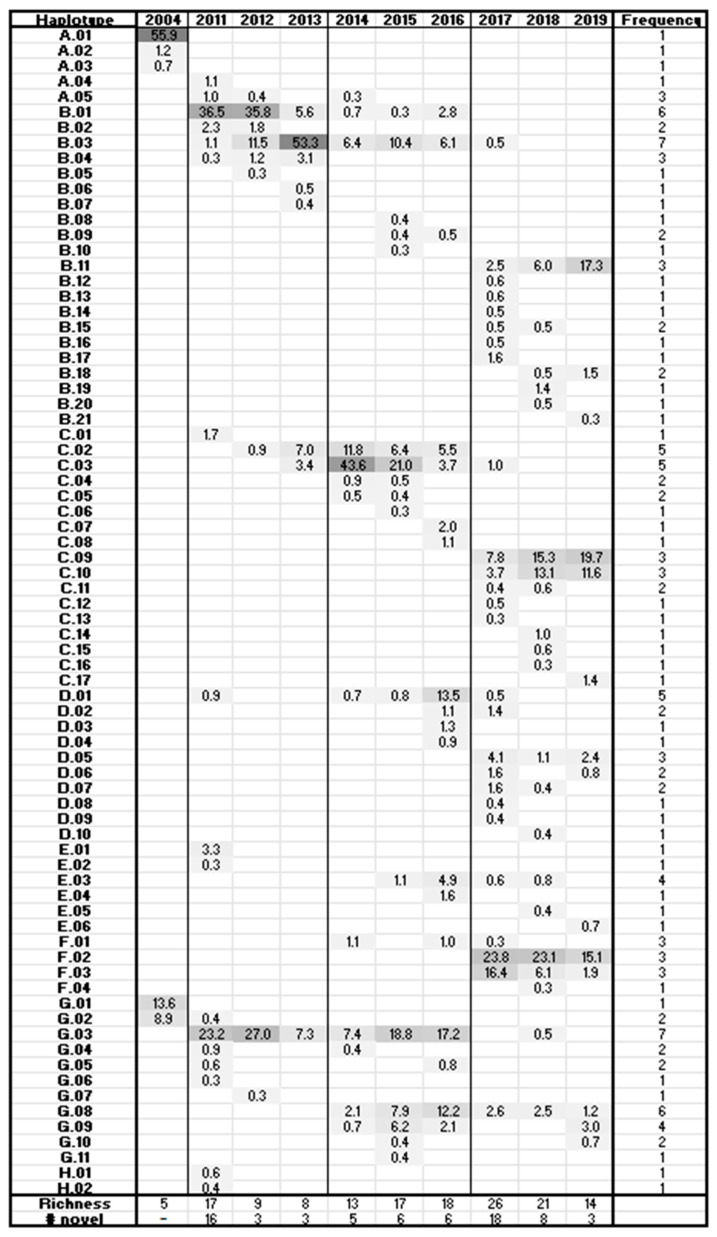
Frequency chart for hypervariable-region haplotypes. Haplotype names are truncated to the clade and order of emergence. Haplotypes are sorted by year and then in order of decreasing frequency. Values indicate the frequency of a given haplotype in each sample.

The HV region showed continuous, complex community transformation (Figure 6). The 2004 community detected by this analysis was composed of two clades, A and G, containing three and two predominant sequences, respectively, with A.01 in the master haplotype position (55.9% of the population). By 2011 the community had diverged from the 2004 haplotypes, and the initial haplotypes had permanently disappeared from the dominant population except for haplotype G.02. Haplotypes A.01 and G.02 were still present in the minority population (<0.3% prevalence) until 2018. During the period 2011–2013, haplotypes B.01, B.03, and G.03 dominated the population, with B.03 increasing steadily as the other two retreated. During this time, the population richness (S) decreased from 17 to 8 haplotypes and the GSI from 0.67 to 0.53. Between 2013 and 2014, a large community shift was accompanied by a leap in richness to 13 and GSI to 0.64. The shift included a transition from master haplotype B.03 (53.3%) toward previous minority members C.02 and C.03, which became dominant at 11.8% and 43.6% of the population, respectively. Despite the other large community changes, haplotype G.03 was maintained at ~7% through the shift. The community composition in years 2014–2016 was largely stagnant with continued high richness, except that the evenness increased from 0.52 to 0.82. A handful of haplotypes slowly rotated through the dominant positions: C.03 dropped to 3.7% in 2016, to be replaced by master sequence G.03 (17.2%), G.08 (12.2%), and D.01 at 13.5%. In 2017, another large shift occurred in which 69% of the haplotypes were novel, with emergent F.02 (23.8%), F.03 (16.4%), and C.09 (7.8%) in the dominant positions. Richness also increased to 26. In the final two years, the 2017 community composition and structure were largely maintained, but with richness decreasing and evenness increasing in 2019. In contrast to the CS region in which the 2013–2014 and 2016–2017 shifts resulted in fewer haplotypes, HV haplotype richness increased in concert with viral load.

### 3.6. Distribution Similarities

**Figure 7:**
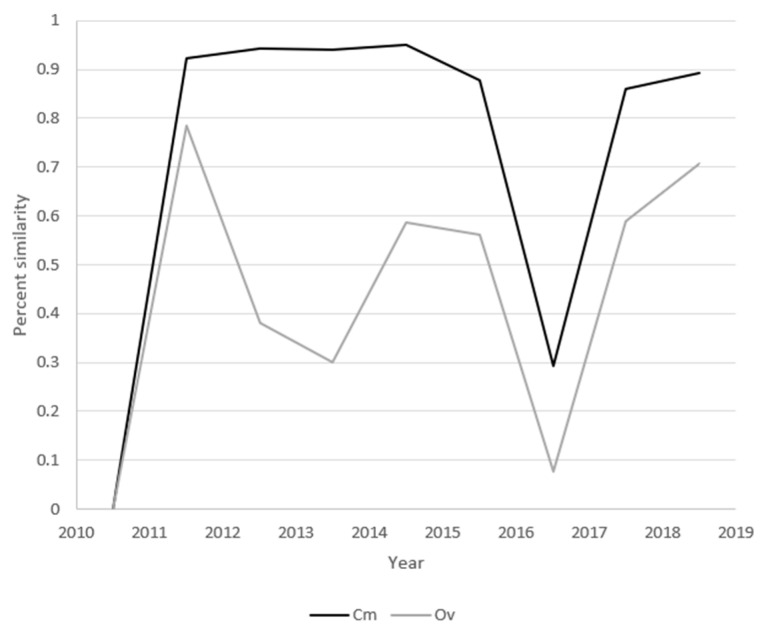
Distribution similarities between sequential samples. Each data point is the diversity value of the two flanking samples. Values for the index of commons (C*_m_*, black) and overlap index (O*_v_*, grey) are overlaid for comparison.

Sequential-sample HV haplotypes were compared using both C*_m_* and O*_v_*. C*_m_* indicated that samples from years 2011–2016 shared >80% of their haplotypes in the years immediately before and immediately after each (Figure 7). Between 2016 and 2017, the community changed abruptly such that <10% of haplotypes were shared. O*_v_* describes the similarity of both common haplotypes and the abundance of these haplotypes between two populations. Strong dips in the O*_v_* similarities between 2013 and 2014 and 2016 and 2017 indicate community reconfigurations. Combined with the high 2013–2014 C*_m_*, the low 2013–2014 O*_v_* supports a community shift from one dominant set of extant haplotypes toward a different set of the same haplotypes. Because both the 2016–2017 C*_m_* and O*_v_* dropped to ≤30%, the second community shift appears to have resulted from the emergence of new dominant haplotypes.

### 3.7. MEME/FUBAR

**Figure 8:**
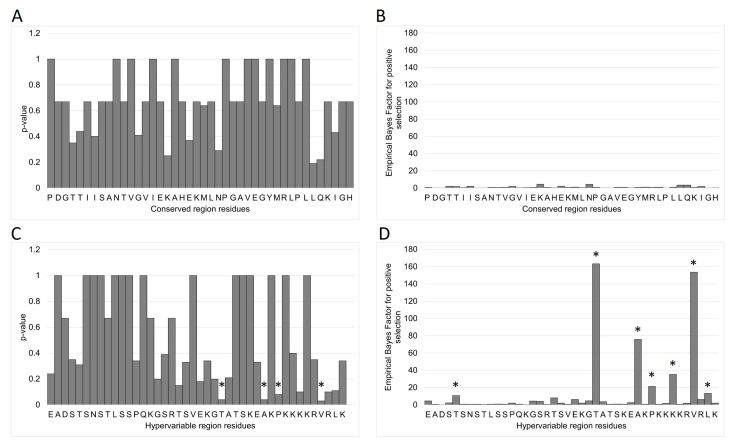
Results from the episodic diversifying selection analyses performed on the MAFFT haplotype alignments. No conserved-locus amino acids were identified as subject to selection by either (**A**) Mixed Effects Model of Evolution (MEME) or (**B**) Fast Unconstrained Bayesian AppRoximation (FUBAR) analyses. MEME identified (**C**) four HV-locus amino acids subject to selection. FUBAR singled out the same four loci (**D**) and three additional ones. The identified amino acids are marked with (*).

The MEME and FUBAR analyses were performed on the MAFFT alignments of all 2004–2019 CS and HV haplotypes to identify sites subject to episodic selection [44]. As described in the phylogenetic analysis above, no CS haplotype was more than 1 nt different from master haplotype I.01; therefore, no amino acid sequence differed from I.01 by more than a maximum of one potential amino acid residue. As expected, the episodic selection algorithms did not detect any CS residues subject to evolutionary pressure (Figure 8A,B). Four amino acids were identified at the HV locus with *p* < 0.1: T25, A31, S33, and L39 (Figure 8C,D). FUBAR identified these same four loci and additionally T5, K36, and L41 with a Bayes Factor > 10 [43].

## 4. Discussion

The data described here provide the first examination of EqPV-H biology and ecology based on the antigenic VP coat protein in a healthy carrier host. When this project was initiated, Divers et al. had proposed that horses testing positive for EqPV-H should be considered persistently infected [3]. To investigate this claim, ten serum samples collected from a single horse over a 16-year period were examined for the current study. Digital PCR quantification of EqPV-H in these samples showed evidence of a persistent infection where the viral load fluctuated with a three-year periodicity (Figure 1). LIPS analysis indicated a low antibody titer in the first sample followed by a persistent high titer, evidence that the first sample was likely obtained shortly after infection. Considering these observations, Illumina miSeq HTS of the 10 samples was performed to further examine the nature of persistent infection.

Because the viral load throughout infection was too low for direct HTS from extracted DNA, the VP gene was PCR-amplified. Extraneously introduced mutations were minimized in the data set by performing the amplification and HTS procedure twice for each sample, allowing false haplotypes to be excluded during the data trimming step. Any impact of artificially introduced haplotypes was further reduced by the comparison of two regions of the VP gene. The first region is a highly conserved locus that corresponds to the CPV capsid GH loop, amino acids 494–536. These residues line the twofold depression and are likely important for capsid assembly [46]. The second region is a hypervariable locus with unknown function, found between the VP2 sequence encoding the 5-fold-axis glycine-rich pore and VP1u PLA2. This HV region also correlates with the adeno-associated virus (AAV) VP1/2 region previously described as “disordered” [47]. It was hypothesized to act as a flexible hinge linking the PLA2 and nuclear localization signals to the highly structured VP2 [48].

The stable CS locus provided a baseline for the examination of the HV region. Evidence from the CS locus indicated that if PCR did impart mutations, these contributions were inconsequential to the analysis. Master haplotype I.01 predominated throughout, while individual minor sequences never exceeded 2.1% of the population. The emergence of new CS haplotypes was not correlated with periodicity, and once introduced to the community, many haplotypes were retained throughout. For example, 2004 haplotypes I.04 and I.08 appeared in 80% and 60% of samples, respectively.

In comparison to the CS locus, continuous evolution at the HV locus was evidenced as haplotype composition and frequency variations, showing a pattern of initial emergence, dominance, and recession with replacement (Figure 4 and Figure 6). Although the initial haplotypes A.01 and G.01 were missing by 2011, related haplotypes had emerged. The disappearance of those vanguard sequences suggests suboptimal fitness for these haplotypes in the new host environment. Haplotypes G.01 and G.02 differed by a single amino acid caused by a point mutation. It is possible that G.02 evolved shortly after infection to increase fitness in the new environment. By 2011 all clades were in evidence, suggesting diversification in response to infection.

Clades A and G displayed different evolutionary strategies, with A mutating rapidly and the progeny maintaining master haplotype status. G maintained a steady, relatively low abundance in the community with a lower mutation rate. While this strategy appears to have been successful for about 13 years, the clade represented a far smaller percent of the haplotypes in the final three years, suggesting a move toward lower fitness. In Figure 4, the samples from clades G and H appear to branch from clade B. The presence of haplotypes G.01 and G.02 in the initial sample combined with the 6 nt difference and 92% confidence of the split between clades H and B show that clade H evolved away from G toward B.

The presence of both clades A and G in the initial 2004 sample implies that either a single event introduced both haplotypes or two consecutive events (superinfection) contributed to the ultimate population. Parvoviruses have been reported to use a strategy of defective interfering particles (DIPs) for superinfection exclusion [49]. Conversely, co-infections of CPV and feline panleukopenia virus (FPV) have been reported in individual cats, suggesting that multiple consecutive infections are possible under some circumstances [50,51]. Horse A cohabited with other EqPV-H-positive animals throughout the 16 years. The examination of haplotype evolution failed to identify any overt evidence for superinfection events. This detail may support the hypothesis of simultaneous infection. Sources of simultaneous infection could be the contamination of shared blood-processing equipment or a single donor hosting a mature EqPV-H population. Haplotypes A.01 and G.01 were 12 nt different from one another. A maximum of 13 nt differences were observed between haplotypes B.20 and F.04, both descended from A.01 after 16 years of HV evolution, leading to a calculated mutation frequency of 4.6 × 10^−3^ mutations/nt/year. Therefore, a single infection event from a sole donor is plausible.

The index of commons (C*_m_*) and overlap index (O*_v_*) analyses identified two different modes of community turnover in two punctuated community shifts (Figure 6 and Figure 7). Combined with the high 2013–2014 C*_m_* value, the low 2013–2014 O*_v_* supports a community shift from one dominant set of extant haplotypes toward a different set of the same haplotypes. Because both the 2016–2017 C*_m_* and O*_v_* dropped to ≤30%, the second community shift appears to have resulted from the emergence of a new but related set of dominant haplotypes. The causes of the community shifts are unknown and outside the scope of this study, but immune pressure has been shown to drive the diversification of viruses such as HIV, HCV, and poliovirus [52]. The relationship between simian immunodeficiency virus (SIV) diversification and immune pressure was elegantly described in 2012 [53].

To better understand the observed population development, MEME and FUBAR analyses were performed on the CS and HV sequence alignments (Figure 8). No positive selection was detected at the CS locus. MEME identified positive selection at four HV amino acids and FUBAR identified three others. Most of the affected residues are in or near the charged lysine-rich C-terminus, which is possibly a nuclear localization signal (NLS) [54,55]. This putative classical monopartite NLS with consensus motif KKKKR is similar to the NLS BC4 (RAKKK) of minute virus of mice (MLM), the porcine parvovirus (PPV) NLS BR5 (KKKAK), and the adeno-associated virus (AAV1) BR3 (PARKR) all found in the genomic VP1u regions [56,57,58]. The MVM BC4 was previously found to be essential for infectivity but was not found to be involved specifically in nuclear transport of VP1 during the infection cycle, while the equivalent bipartite BR5 of PPV was sufficient for nuclear import and the AAV1 BR3 was essential [58,59]. The importance of the NLS in the infection process may explain why all observed EqPV-H motif mutations were lysine to arginine, maintaining the positive charge of the locus. Alignments of AAV1 BR3 sequences indicate that this variety exists in nature. A similar variety has been described for the NLS located in hypervariable regions of pathogens such as HCV, HIV, and Newcastle Disease Virus [60,61,62].

The EqPV-H HV region may contain both a nuclear localization sequence and an immunogenically important target. It was previously reported that the CPV BC4-equivalent epitope RPPPHIFINLAKKKKAGA generated antibodies with strong neutralizing capacity [63,64,65]. Antibody responses to the PPV BR5 have also been observed [59,64,66]. These observations are curious; evidence suggests that the VP1u of most parvoviruses is externalized after contact with the acidic endosome environment; therefore, the generation of neutralizing antibody to VP1u is unexplained [12]. The large (227 amino acids) VP1u of human parvovirus B19 is a target for neutralizing antibody and is thought to be moved to the exterior of the capsid after initial receptor binding but before capsid internalization [12,67,68,69]. It was also reported that the N-terminus of VP2 resides outside the B19 capsid at the 5-fold axis prior to infection, suggesting that the VP1u may therefore also be outside [70]. The EqPV-H VP1u is unusually large (367 amino acids) and so is likely exposed on the capsid surface.

Further work is necessary to determine whether the observed high viral mutation rate contributes to persistent EqPVH infection. The observed characteristics of a bottleneck at infection, high mutation rate, population expansion and diversification, punctuated evolution, and tendency toward evenness described here are all facets of the QS concept [52]. In addition, further examination of sequential samples obtained from other horses could determine whether these ecological traits are typical for EqPV-H. Such studies should provide insight into viral evolution during the early stages of infection, data missing from this study [53,71]. The analysis of samples from horses cohabitating with horse A may also provide insight into virus transmission and dynamics within a premises.

## Figures and Tables

**Table 1:** Statistics from the high-throughput sequencing (HTS) data at the conserved (CS) locus.

Sample	Total # Reads From 2 HTS Runs	# Reads Mapped to CS Locus	# Unique Haplotypes Before Trimming	Haplotype Richness (*S*)	Gini–Simpson Index (GSI)	Evenness (J)
2004	4,358,836	58,460	1835	8	0.37	0.34
2011	4,091,698	77,565	1999	7	0.06	0.10
2012	5,450,603	68,591	1758	11	0.09	0.13
2013	3,797,702	39,964	1281	9	0.08	0.12
2014	4,103,750	52,498	1359	7	0.05	0.09
2015	3,982,446	51,411	1546	12	0.11	0.14
2016	3,067,378	34,916	1176	11	0.12	0.15
2017	3,927,682	42,277	1237	7	0.05	0.08
2018	3,664,280	40,133	1214	10	0.07	0.11
2019	4,569,344	49,490	1558	11	0.13	0.17
Total	41,013,719	515,305	NA ^‡^	38 *	NA	NA
Average	4,101,372	51,531	1496	9	NA	NA
StDev	623,835	13,529	290	2	NA	NA
CV	14	25	18	20	NA	NA

* Haplotypes detected in multiple samples counted only once; ^‡^ NA, not applicable.

**Table 2:** Statistics from the high-throughput sequencing (HTS) data at the hypervariable (HV) locus.

Sample	Total # Reads From 2 HTS Runs	# Reads Mapped to HV Locus	# Unique Haplotypes Before Trimming	Haplotype Richness (*S*)	Gini–Simpson Index (GSI)	Evenness (J)
2004	4,358,836	54,232	1932	5	0.48	0.43
2011	4,091,698	61,031	3759	17	0.67	0.51
2012	5,450,148	69,370	2842	9	0.66	0.51
2013	3,797,702	43,201	2005	8	0.53	0.57
2014	4,103,750	50,979	2831	13	0.64	0.52
2015	3,982,446	48,838	3137	17	0.82	0.67
2016	3,067,378	35,448	2814	18	0.87	0.82
2017	3,927,682	39,047	3342	25	0.98	0.69
2018	3,664,280	40,082	3063	21	0.82	0.67
2019	4,556,164	52,073	3187	14	0.82	0.72
Total	41,000,084	494,301	NA ^‡^	95 *	NA	NA
*Average*	4,100,008	49,430	2891	15	NA	NA
StDev	660,640	10,512	563	6	NA	NA
CV	14	20	18	40	NA	NA

* Haplotypes detected in multiple samples counted only once; ^‡^ NA, not applicable.

## Data Availability

The original contributions presented in this study are included in the article/Supplementary Materials. Further inquiries can be directed to the corresponding author(s). The raw data supporting the conclusions of this article will be made available by the authors on request.

## Supplementary Materials

The following supporting information can be downloaded at https://www.mdpi.com/article/10.3390/v17070947/s1, Section S1: GenBank accession numbers of sequences used to identify the VP hypervariable region; Section S2: Alignment of VP sequences listed in S1 showing the location of the HV and CS regions; Section S3: CS fasta sequences; Section S4: HV fasta sequences.
