# Supplementary material for: Equine Parvovirus-Hepatitis Population Dynamics in a Single Horse over 16 Years
Source: Viruses. 2025 Jul 4;17(7):947. doi: 10.3390/v17070947 (PMC12299937; doi:10.3390/v17070947)
Supplement: Supplementary file 1 [file viruses-17-00947-s001.zip › viruses-3654876-supplementary.pdf]

Equine parvovirus- hepatitis population dynamics in long-term infection

Alexandra J. Scupham[1]

[1]Animal and Plant Health Inspection Service, Center for Veterinary Biologics, Ames, IA, USA.
Corresponding author: Alexandra J. Scupham, Animal and Plant Health Inspection Service, Center for Veterinary Biologics, 1920 Dayton Ave, Ames, IA 50010, USA. alexandra.scupham@usda.gov

Supplemental materials:

Section S1. GenBank accession numbers of sequences used to identify the VP hypervariable region.

MW256660

MN218584

MW256662

MW256663

MN218583

MW256661

MH500792

MH500791

MH500790

MN218587

MH500788

MH500789

MN218585

MH500787

MN218586

MG136722

Section S2. Alignment of VP sequences listed in S1 showing the location of the HV and CS regions.

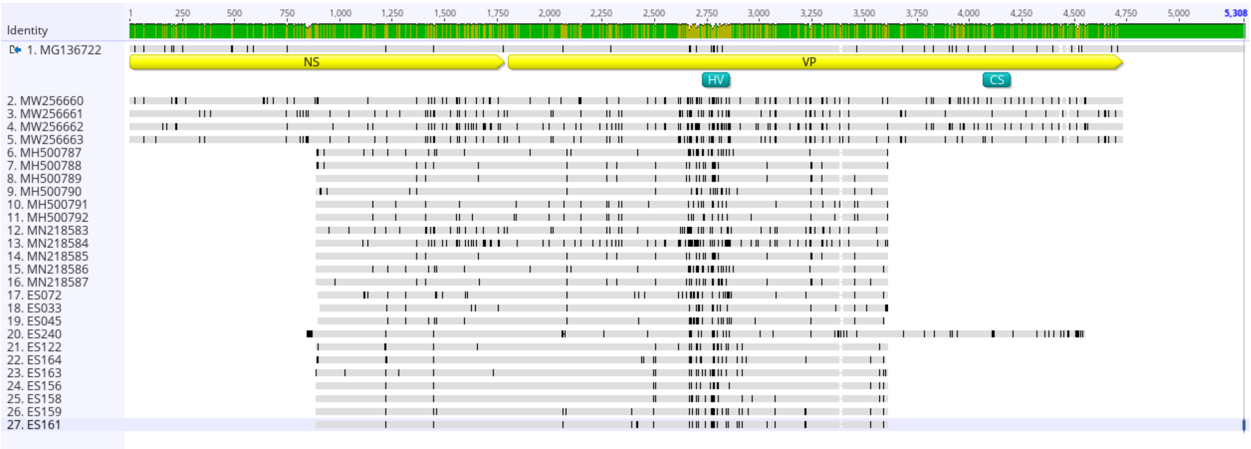

Section S3. CS fasta sequences.

>55-04_CS_36696_I.1

CCCGATGGTACAACCATCATTTCCGCCAACACAGTGGGGGTAATAGAAAAGGCCCATGAGAAAATGTTAAATCCC
GGGGCTGTGGAGGGATACATGCGCCTTCCCCTTCTGCAGAAAATCGGGCAT

>55-04_CS_9108_I.2

CCCGATGGTACAACCATCATTTCCGCCAACACAGTGGGGGTAATAGAAAAGGCCCATGAGAAAATGTTAAATCCC
GGGGCTGTGGAGGGATACATGCGCCTTCCCCTTCTGCAGAAAATCGGACAT

>55-04_CS_407_I.3

CCCGATGGTACAACCATCATTTCCGCCAACACAGTGGGGGTAATAGAAAAGGCCCATGAGAAGATGTTAAATCCC
GGGGCTGTGGAGGGATACATGCGCCTTCCCCTTCTGCAGAAAATCGGGCAT

>55-04_CS_347_I.4

CCCGATGGTACAACCATCATTTCCGCCAACACAGTGGGGGTAATAGAAAGGGCCCATGAGAAAATGTTAAATCCC
GGGGCTGTGGAGGGATACATGCGCCTTCCCCTTCTGCAGAAAATCGGGCAT

>55-04_CS_267_I.5

CCCGATGGTACAACCATCATTTCCGCTAACACAGTGGGGGTAATAGAAAAGGCCCATGAGAAAATGTTAAATCCC
GGGGCTGTGGAGGGATACATGCGCCTTCCCCTTCTGCAGAAAATCGGGCAT

>55-04_CS_251_I.6

CCCGATGGTGCAACCATCATTTCCGCCAACACAGTGGGGGTAATAGAAAAGGCCCATGAGAAAATGTTAAATCCC
GGGGCTGTGGAGGGATACATGCGCCTTCCCCTTCTGCAGAAAATCGGGCAT

>55-04_CS_247_I.7

CCCGATGGTACAACCACCATTTCCGCCAACACAGTGGGGGTAATAGAAAAGGCCCATGAGAAAATGTTAAATCCC
GGGGCTGTGGAGGGATACATGCGCCTTCCCCTTCTGCAGAAAATCGGGCAT

>55-04_CS_184_I.8

CCCGATGGTACAACCATCATTTCCGCCAACACAGTGGGGGTAATAGAAAAGGCCCATGGGAAAATGTTAAATCCC
GGGGCTGTGGAGGGATACATGCGCCTTCCCCTTCTGCAGAAAATCGGGCAT

>55-11_CS_61033_I.1

CCCGATGGTACAACCATCATTTCCGCCAACACAGTGGGGGTAATAGAAAAGGCCCATGAGAAAATGTTAAATCCC
GGGGCTGTGGAGGGATACATGCGCCTTCCCCTTCTGCAGAAAATCGGGCAT

>55-11_CS_433_I.9

CCCGATGGTACAACCATCATTTCCGCCAACACAGTGAGGGTAATAGAAAAGGCCCATGAGAAAATGTTAAATCCC
GGGGCTGTGGAGGGATACATGCGCCTTCCCCTTCTGCAGAAAATCGGGCAT

>55-11_CS_398_I.10

CCCGATGGTACAACCATCATTTCCGCCAACGCAGTGGGGGTAATAGAAAAGGCCCATGAGAAAATGTTAAATCCC
GGGGCTGTGGAGGGATACATGCGCCTTCCCCTTCTGCAGAAAATCGGGCAT

>55-11_CS_354_I.11

CCCGATGGTACAACCATCATTTCCGCCAACACAGTGGGGGTAATAGAAAAGGCCCATGAGAAAATGTTAAATCCC
GGGGCTGTGGAGGGATACATGCGCCTTCCCCTTCTGCAGAAAATCGGGCAC

>55-11_CS_292_I.8

CCCGATGGTACAACCATCATTTCCGCCAACACAGTGGGGGTAATAGAAAAGGCCCATGGGAAAATGTTAAATCCC
GGGGCTGTGGAGGGATACATGCGCCTTCCCCTTCTGCAGAAAATCGGGCAT

>55-11_CS_274_I.12

CCCGATGGTACAACCATCATTTCCGCCAACACAGTGGGGGTAATAGAAAAGGCCCATGAGAAAATGTTAAATCCC
GGGGCTGTGGAGGGGTACATGCGCCTTCCCCTTCTGCAGAAAATCGGGCAT

>55-11_CS_236_I.4

CCCGATGGTACAACCATCATTTCCGCCAACACAGTGGGGGTAATAGAAAGGGCCCATGAGAAAATGTTAAATCCC
GGGGCTGTGGAGGGATACATGCGCCTTCCCCTTCTGCAGAAAATCGGGCAT

>55-12_CS_54075_I.1

CCCGATGGTACAACCATCATTTCCGCCAACACAGTGGGGGTAATAGAAAAGGCCCATGAGAAAATGTTAAATCCC
GGGGCTGTGGAGGGATACATGCGCCTTCCCCTTCTGCAGAAAATCGGGCAT

>55-12_CS_561_I.13

CCCGATGGTACAACCATCATTTCCGCCAACACAGTGGGGGTAATAGAAAAGGCCCATGAGAAAATGTTAAATCCC
GGGGCTGTGGAGGGATACATGCGCCTTCCCCTTCTGCAGAAGATCGGGCAT

>55-12_CS_328_I.14

CCCGATGGTACAACCATCATTTCCGCCAACACAGTGGGGGTAATAGAAAAGGCCCACGAGAAAATGTTAAATCCC
GGGGCTGTGGAGGGATACATGCGCCTTCCCCTTCTGCAGAAAATCGGGCAT

>55-12_CS_274_I.15

CCCGATGGTACAGCCATCATTTCCGCCAACACAGTGGGGGTAATAGAAAAGGCCCATGAGAAAATGTTAAATCCC
GGGGCTGTGGAGGGATACATGCGCCTTCCCCTTCTGCAGAAAATCGGGCAT

>55-12_CS_254_I.4

CCCGATGGTACAACCATCATTTCCGCCAACACAGTGGGGGTAATAGAAAGGGCCCATGAGAAAATGTTAAATCCC
GGGGCTGTGGAGGGATACATGCGCCTTCCCCTTCTGCAGAAAATCGGGCAT

>55-12_CS_252_I.16

CCCGATGGTACAACCATCATTTCCGCCAACACAGTGGGGGTAATAGAAAAGGCCCATGAGAAAATGTTAAATCCC
GGGGCTGTGGAGGGATACGTGCGCCTTCCCCTTCTGCAGAAAATCGGGCAT

>55-12_CS_238_I.17

CCCGATGGTACAACCATCATTTCCGCCAACACAGTGGGGGTAATAGGAAAGGCCCATGAGAAAATGTTAAATCCC
GGGGCTGTGGAGGGATACATGCGCCTTCCCCTTCTGCAGAAAATCGGGCAT

>55-12_CS_225_I.18

CCCGATGGTACAACCATCATTTCCGCCAACACAGTGGGGGTAATAGAAGAGGCCCATGAGAAAATGTTAAATCCC
GGGGCTGTGGAGGGATACATGCGCCTTCCCCTTCTGCAGAAAATCGGGCAT

>55-12_CS_218_I.19

CCCGATGGTACAACCATCATTTCCGCCAACACAGTGGGGGTAATAGAAAAGGCCCATGAGGAAATGTTAAATCCC
GGGGCTGTGGAGGGATACATGCGCCTTCCCCTTCTGCAGAAAATCGGGCAT

>55-12_CS_209_I.8

CCCGATGGTACAACCATCATTTCCGCCAACACAGTGGGGGTAATAGAAAAGGCCCATGGGAAAATGTTAAATCCC
GGGGCTGTGGAGGGATACATGCGCCTTCCCCTTCTGCAGAAAATCGGGCAT

>55-12_CS_209_I.20

CCCGATGGTACAACCATCATTTCCGCCAACACAGTGGGGGTAATAGAAAAGGCCCATGAGAAAATGTTAAGTCCC
GGGGCTGTGGAGGGATACATGCGCCTTCCCCTTCTGCAGAAAATCGGGCAT

>55-13_CS_31248_I.1

CCCGATGGTACAACCATCATTTCCGCCAACACAGTGGGGGTAATAGAAAAGGCCCATGAGAAAATGTTAAATCCC
GGGGCTGTGGAGGGATACATGCGCCTTCCCCTTCTGCAGAAAATCGGGCAT

>55-13_CS_287_I.21

CCCGATGGTACAACCATCATTTCCGCCAACACAGTGGGGGTAATAGAAAAGGCCCATGAGAAAATGTTAAATCCC
GGAGCTGTGGAGGGATACATGCGCCTTCCCCTTCTGCAGAAAATCGGGCAT

>55-13_CS_272_I.22

CCCGATGGTACAACCATCGTTTCCGCCAACACAGTGGGGGTAATAGAAAAGGCCCATGAGAAAATGTTAAATCCC
GGGGCTGTGGAGGGATACATGCGCCTTCCCCTTCTGCAGAAAATCGGGCAT

>55-13_CS_172_I.4

CCCGATGGTACAACCATCATTTCCGCCAACACAGTGGGGGTAATAGAAAGGGCCCATGAGAAAATGTTAAATCCC
GGGGCTGTGGAGGGATACATGCGCCTTCCCCTTCTGCAGAAAATCGGGCAT

>55-13_CS_138_I.23

CCCGATGGTACAACCATCATTTCCGCCAACACAGTGGGGGTAATAGGAAAGGCCCATGAGAAAATGTTAAATCCC
GGGGCTGTGGAGGGATACATGCGCCTTCCCCTTCTGCAGAAAATCGGGCAT

>55-13_CS_128_I.24

CCCGATGGTACAGCCATCATTTCCGCCAACACAGTGGGGGTAATAGAAAAGGCCCATGAGAAAATGTTAAATCCC
GGGGCTGTGGAGGGATACATGCGCCTTCCCCTTCTGCAGAAAATCGGGCAT

>55-13_CS_124_I.25

CCCGATGGTACAACCATCATTTCCGCCAACACAGTGGGGGTAATAGAAAAGGCCCATGAGAAAATGTTAAATCCC
GGGGCTGTGGAGGGATACATGCGCCTTCCCCTTCTGCAGAGAATCGGGCAT

>55-13_CS_124_I.26

CCCGATGGTACAACCATCATTTCCGCCAACACAGTGGGGGTAATAGAAAAGGCCCATGAGGAAATGTTAAATCCC
GGGGCTGTGGAGGGATACATGCGCCTTCCCCTTCTGCAGAAAATCGGGCAT

>55-13_CS_120_I.27

CCCGATGGTACAACCATCATTTCCGCCAACACAGTGGGGGTAATAGAAAAGGCCCATGAGAAAATGTTAAATCCC
GGGGCTGTGGAGGGGTACATGCGCCTTCCCCTTCTGCAGAAAATCGGGCAT

>55-14_CS_41984_I.1

CCCGATGGTACAACCATCATTTCCGCCAACACAGTGGGGGTAATAGAAAAGGCCCATGAGAAAATGTTAAATCCC
GGGGCTGTGGAGGGATACATGCGCCTTCCCCTTCTGCAGAAAATCGGGCAT

>55-14_CS_297_I.28

CCCGATGGTACAACCATCATTTCCGCCAACACAGTGGGGGTCATAGAAAAGGCCCATGAGAAAATGTTAAATCCC
GGGGCTGTGGAGGGATACATGCGCCTTCCCCTTCTGCAGAAAATCGGGCAT

>55-14_CS_185_I.29

CCCGATGGTACAGCCATCATTTCCGCCAACACAGTGGGGGTAATAGAAAAGGCCCATGAGAAAATGTTAAATCCC
GGGGCTGTGGAGGGATACATGCGCCTTCCCCTTCTGCAGAAAATCGGGCAT

>55-14_CS_173_I.30

CCCGATGGTACAACCATCATTTCCGCCAACACAGTGGGGGTAATAGAAAAGGCCCATGAGAAAATGTTAAATCCC
GGGGCTGTGGAGGGATACATGCGCCTTCCCCTTCTGCGGAAAATCGGGCAT

>55-14_CS_173_I.31

CCCGATGGTACAACCATCATTTCCGCCAACACGGTGGGGGTAATAGAAAAGGCCCATGAGAAAATGTTAAATCCC
GGGGCTGTGGAGGGATACATGCGCCTTCCCCTTCTGCAGAAAATCGGGCAT

>55-14_CS_165_I.4

CCCGATGGTACAACCATCATTTCCGCCAACACAGTGGGGGTAATAGAAAGGGCCCATGAGAAAATGTTAAATCCC
GGGGCTGTGGAGGGATACATGCGCCTTCCCCTTCTGCAGAAAATCGGGCAT

>55-14_CS_165_I.32

CCCGATGGTACAACCATCATTTCCGCCAACACAGTGGGGGTAATAGGAAAGGCCCATGAGAAAATGTTAAATCCC
GGGGCTGTGGAGGGATACATGCGCCTTCCCCTTCTGCAGAAAATCGGGCAT

>55-15_CS_39906_I.1

CCCGATGGTACAACCATCATTTCCGCCAACACAGTGGGGGTAATAGAAAAGGCCCATGAGAAAATGTTAAATCCC
GGGGCTGTGGAGGGATACATGCGCCTTCCCCTTCTGCAGAAAATCGGGCAT

>55-15_CS_358_I.33

CCCGATGGTACAACCATCATTTCCGCCAACACAGTGGGGGTCATAGAAAAGGCCCATGAGAAAATGTTAAATCCC
GGGGCTGTGGAGGGATACATGCGCCTTCCCCTTCTGCAGAAAATCGGGCAT

>55-15_CS_324_I.34

CCCGATGGTACAACCATCATTTCCGCCAACACAGTGGGGGTAATAGAAAAGGCCCATGAGAAAATGCTAAATCCC
GGGGCTGTGGAGGGATACATGCGCCTTCCCCTTCTGCAGAAAATCGGGCAT

>55-15_CS_235_I.8

CCCGATGGTACAACCATCATTTCCGCCAACACAGTGGGGGTAATAGAAAAGGCCCATGGGAAAATGTTAAATCCC
GGGGCTGTGGAGGGATACATGCGCCTTCCCCTTCTGCAGAAAATCGGGCAT

>55-15_CS_201_I.35

CCCGATGGTACAACCATCATTTCCGCCAACACAGTGGGGGTAATAGAAAAGGCCCATGAGAAAATGTTAAATCCC
GGAGCTGTGGAGGGATACATGCGCCTTCCCCTTCTGCAGAAAATCGGGCAT

>55-15_CS_190_I.36

CCCGATGGTACAACCATCATTTCCGCCAACACAGTGGGGGTAATAGAAAAGGCCCATGAGAAAATGTTAAATCCC
GGGGCCGTGGAGGGATACATGCGCCTTCCCCTTCTGCAGAAAATCGGGCAT

>55-15_CS_180_I.4

CCCGATGGTACAACCATCATTTCCGCCAACACAGTGGGGGTAATAGAAAGGGCCCATGAGAAAATGTTAAATCCC
GGGGCTGTGGAGGGATACATGCGCCTTCCCCTTCTGCAGAAAATCGGGCAT

>55-15_CS_170_I.37

CCCGATGGTACAGCCATCATTTCCGCCAACACAGTGGGGGTAATAGAAAAGGCCCATGAGAAAATGTTAAATCCC
GGGGCTGTGGAGGGATACATGCGCCTTCCCCTTCTGCAGAAAATCGGGCAT

>55-15_CS_165_I.38

CCCGATGGTACAACCATCATTTCCGCCAACACAGTGGGGGTAATAGAAAAGGCCCATGAGAAAATGTTAAATCCC
GGGGCTGTGGAGGGATACATGCGCCTTCCCCTTCTGCAGAAAGTCGGGCAT

>55-15_CS_162_I.39

CCCGATGGTACAACCATCATTTCCGCCAACACAGTGGGGGTAATAGGAAAGGCCCATGAGAAAATGTTAAATCCC
GGGGCTGTGGAGGGATACATGCGCCTTCCCCTTCTGCAGAAAATCGGGCAT

>55-15_CS_162_I.40

CCCGATGGTACAACCATCATTTCCGCCAACACGGTGGGGGTAATAGAAAAGGCCCATGAGAAAATGTTAAATCCC
GGGGCTGTGGAGGGATACATGCGCCTTCCCCTTCTGCAGAAAATCGGGCAT

>55-15_CS_155_I.41

CCCGATGGTACAACCATCATTTCCGCCAACACAGTGGGGGTAATAGAGAAGGCCCATGAGAAAATGTTAAATCCC
GGGGCTGTGGAGGGATACATGCGCCTTCCCCTTCTGCAGAAAATCGGGCAT

>55-16_CS_26990_I.1

CCCGATGGTACAACCATCATTTCCGCCAACACAGTGGGGGTAATAGAAAAGGCCCATGAGAAAATGTTAAATCCC
GGGGCTGTGGAGGGATACATGCGCCTTCCCCTTCTGCAGAAAATCGGGCAT

>55-16_CS_727_I.42

CCCGATGGTACAACCATTATTTCCGCCAACACAGTGGGGGTAATAGAAAAGGCCCATGAGAAAATGTTAAATCCC
GGGGCTGTGGAGGGATACATGCGCCTTCCCCTTCTGCAGAAAATCGGGCAT

>55-16_CS_154_I.43

CCCGATGGTACAACCATCATTTCCGCCAACACAGTGGGGGTAATAGAAAAGGCCCATGAGAAAGTGTTAAATCCC
GGGGCTGTGGAGGGATACATGCGCCTTCCCCTTCTGCAGAAAATCGGGCAT

>55-16_CS_148_I.44

CCCGATGGTACAACCATCATTTCCGCCAACACAGTGGGGGTAATAGAAAAGGCCCATGAGAAAATGTTAAATCCC
GGGGCTGTGGAGGGGTACATGCGCCTTCCCCTTCTGCAGAAAATCGGGCAT

>55-16_CS_121_I.45

CCCGATGGTACAGCCATCATTTCCGCCAACACAGTGGGGGTAATAGAAAAGGCCCATGAGAAAATGTTAAATCCC
GGGGCTGTGGAGGGATACATGCGCCTTCCCCTTCTGCAGAAAATCGGGCAT

>55-16_CS_115_I.46

CCCGATGGTACAACCATCATTTCCGCCAACACGGTGGGGGTAATAGAAAAGGCCCATGAGAAAATGTTAAATCCC
GGGGCTGTGGAGGGATACATGCGCCTTCCCCTTCTGCAGAAAATCGGGCAT

>55-16_CS_114_I.8

CCCGATGGTACAACCATCATTTCCGCCAACACAGTGGGGGTAATAGAAAAGGCCCATGGGAAAATGTTAAATCCC
GGGGCTGTGGAGGGATACATGCGCCTTCCCCTTCTGCAGAAAATCGGGCAT

>55-16_CS_111_I.47

CCCGATGGTACAACCATCATTTCCGCCAACACAGTGGGGGTAATAGAAAAGGCCCATGAGAAAATGTTAAATCCC
GGGGCTGTGGAGGGATACATGCGCCTTCCCCTTCTGCGGAAAATCGGGCAT

>55-16_CS_110_I.6

CCCGATGGTGCAACCATCATTTCCGCCAACACAGTGGGGGTAATAGAAAAGGCCCATGAGAAAATGTTAAATCCC
GGGGCTGTGGAGGGATACATGCGCCTTCCCCTTCTGCAGAAAATCGGGCAT

>55-16_CS_109_I.48

CCCGATGGTACAACCATCATTTCCGCCAACACAGTGGGGGTAATAGGAAAGGCCCATGAGAAAATGTTAAATCCC
GGGGCTGTGGAGGGATACATGCGCCTTCCCCTTCTGCAGAAAATCGGGCAT

>55-16_CS_109_I.3

CCCGATGGTACAACCATCATTTCCGCCAACACAGTGGGGGTAATAGAAAAGGCCCATGAGAAGATGTTAAATCCC
GGGGCTGTGGAGGGATACATGCGCCTTCCCCTTCTGCAGAAAATCGGGCAT

>55-17_CS_33810_I.1

CCCGATGGTACAACCATCATTTCCGCCAACACAGTGGGGGTAATAGAAAAGGCCCATGAGAAAATGTTAAATCCC
GGGGCTGTGGAGGGATACATGCGCCTTCCCCTTCTGCAGAAAATCGGGCAT

>55-17_CS_179_I.49

CCCGATGGTACAACCATCATTTCCGCCAACACAGTGGGGGTAATAGAAAAGGCCCATGAGAAAATGTTAAATCCC
GGGGCTGTGGAGGGATACATGCGCCTTCCCCTTCCGCAGAAAATCGGGCAT

>55-17_CS_136_I.50

CCCGACGGTACAACCATCATTTCCGCCAACACAGTGGGGGTAATAGAAAAGGCCCATGAGAAAATGTTAAATCCC
GGGGCTGTGGAGGGATACATGCGCCTTCCCCTTCTGCAGAAAATCGGGCAT

>55-17_CS_135_I.51

CCCGATGGTACAACCATCATTTCCGCCAACACAGTGGGGGTAATAGAAGAGGCCCATGAGAAAATGTTAAATCCC
GGGGCTGTGGAGGGATACATGCGCCTTCCCCTTCTGCAGAAAATCGGGCAT

>55-17_CS_134_I.52

CCCGATGGTACAACCATCATTTCCGCCAACACAGTGGGGGTAATAGAAAAGGCCCATGAGAAAATGTTAAGTCCC
GGGGCTGTGGAGGGATACATGCGCCTTCCCCTTCTGCAGAAAATCGGGCAT

>55-17_CS_134_I.53

CCCGATGGTACAACCATCATTTCTGCCAACACAGTGGGGGTAATAGAAAAGGCCCATGAGAAAATGTTAAATCCC
GGGGCTGTGGAGGGATACATGCGCCTTCCCCTTCTGCAGAAAATCGGGCAT

>55-17_CS_130_I.54

CCCGATGGCACAACCATCATTTCCGCCAACACAGTGGGGGTAATAGAAAAGGCCCATGAGAAAATGTTAAATCCC
GGGGCTGTGGAGGGATACATGCGCCTTCCCCTTCTGCAGAAAATCGGGCAT

>55-18_CS_31817_I.1

CCCGATGGTACAACCATCATTTCCGCCAACACAGTGGGGGTAATAGAAAAGGCCCATGAGAAAATGTTAAATCCC
GGGGCTGTGGAGGGATACATGCGCCTTCCCCTTCTGCAGAAAATCGGGCAT

>55-18_CS_201_I.4

CCCGATGGTACAACCATCATTTCCGCCAACACAGTGGGGGTAATAGAAAGGGCCCATGAGAAAATGTTAAATCCC
GGGGCTGTGGAGGGATACATGCGCCTTCCCCTTCTGCAGAAAATCGGGCAT

>55-18_CS_147_I.55

CCCGATGGTACAACCATCATTTCCGCCAACACAGTGGGGGTAATAGAAAAGGCCCATGAGAAAATGTTAAATCCC
GGGGCTGTGGAGGGATACATGCGCCTTCCCCTTCTGCGGAAAATCGGGCAT

>55-18_CS_142_I.56

CCCGATGGTACAGCCATCATTTCCGCCAACACAGTGGGGGTAATAGAAAAGGCCCATGAGAAAATGTTAAATCCC
GGGGCTGTGGAGGGATACATGCGCCTTCCCCTTCTGCAGAAAATCGGGCAT

>55-18_CS_138_I.57

CCCGATGGTACAACCATCATTTCCGCCAACGCAGTGGGGGTAATAGAAAAGGCCCATGAGAAAATGTTAAATCCC
GGGGCTGTGGAGGGATACATGCGCCTTCCCCTTCTGCAGAAAATCGGGCAT

>55-18_CS_130_I.58

CCCGATGGTACAACCATCATTTCCGCCAACACAGTGGGGGTAATAGAAAAGGCCCATGAGAAAATGTTAAATCCC
GGGGCTGTGGAGGGGTACATGCGCCTTCCCCTTCTGCAGAAAATCGGGCAT

>55-18_CS_130_I.59

CCCGATGGTACAACCATCATTTCCGCCAACACAGTGGGGGTAATAGAAAAGGCCCATGAGGAAATGTTAAATCCC
GGGGCTGTGGAGGGATACATGCGCCTTCCCCTTCTGCAGAAAATCGGGCAT

>55-18_CS_128_I.60

CCCGATGGTACAACCATCATTTCCGCCAACACAGTGGGGGTAATAGAAAAGGCCCATGAGAAAATGTTAAATCCC
GGGGCTGTGGAGGGATACATGCGCCTTCCCCTTCTGCAGAAAGTCGGGCAT

>55-18_CS_128_I.61

CCCGATGGTACAACCATCATTTCCGCCAACACAGTGGGGGTAATAGGAAAGGCCCATGAGAAAATGTTAAATCCC
GGGGCTGTGGAGGGATACATGCGCCTTCCCCTTCTGCAGAAAATCGGGCAT

>55-18_CS_123_I.62

CCCGATGGTACAACCATCATTTCCGCCAACACAGTGGGGGTAATAGAAAAGGCCCATGAGAAAATGTTAAATCCC
GGGGCTGTGGAGGGATACATGCGCCTTCCCCTTCTGCAGAAGATCGGGCAT

>55-19_CS_37467_I.1

CCCGATGGTACAACCATCATTTCCGCCAACACAGTGGGGGTAATAGAAAAGGCCCATGAGAAAATGTTAAATCCC
GGGGCTGTGGAGGGATACATGCGCCTTCCCCTTCTGCAGAAAATCGGGCAT

>55-19_CS_793_I.5

CCCGATGGTACAACCATCATTTCCGCTAACACAGTGGGGGTAATAGAAAAGGCCCATGAGAAAATGTTAAATCCC
GGGGCTGTGGAGGGATACATGCGCCTTCCCCTTCTGCAGAAAATCGGGCAT

>55-19_CS_507_I.8

CCCGATGGTACAACCATCATTTCCGCCAACACAGTGGGGGTAATAGAAAAGGCCCATGGGAAAATGTTAAATCCC
GGGGCTGTGGAGGGATACATGCGCCTTCCCCTTCTGCAGAAAATCGGGCAT

>55-19_CS_328_I.4

CCCGATGGTACAACCATCATTTCCGCCAACACAGTGGGGGTAATAGAAAGGGCCCATGAGAAAATGTTAAATCCC
GGGGCTGTGGAGGGATACATGCGCCTTCCCCTTCTGCAGAAAATCGGGCAT

>55-19_CS_189_I.63

CCCGATGGTACAACCATCATTTCCGCCAACACAGTGGGGGTAATAGAAAAGGCCCATGAGAAAATGTTAAATCCC
GGGGCTGTGGAGGGATACATGCGCCTTCCCCTTCTGCAGAAGATCGGGCAT

>55-19_CS_185_I.64

CCCGATGGTACAACCATCATTTCCGCCAACACAGTGGGGGTAATAGAAAAGGCCCATGAGGAAATGTTAAATCCC
GGGGCTGTGGAGGGATACATGCGCCTTCCCCTTCTGCAGAAAATCGGGCAT

>55-19_CS_182_I.65

CCCGATGGTACAACCATCATTTCCGCCAACACAGTGGGGGTAATAGGAAAGGCCCATGAGAAAATGTTAAATCCC
GGGGCTGTGGAGGGATACATGCGCCTTCCCCTTCTGCAGAAAATCGGGCAT

>55-19_CS_157_I.66

CCCGATGGTACAACCATCATTTCCGCCAACACAGTGGGGGTAATAGAAAAGGCCCATGAGAAAATGTTAGATCCC
GGGGCTGTGGAGGGATACATGCGCCTTCCCCTTCTGCAGAAAATCGGGCAT

>55-19_CS_155_I.67

CCCGATGGTACAACCATCATTTCCGCCAACGCAGTGGGGGTAATAGAAAAGGCCCATGAGAAAATGTTAAATCCC
GGGGCTGTGGAGGGATACATGCGCCTTCCCCTTCTGCAGAAAATCGGGCAT

>55-19_CS_153_I.68

CCCGATGGTACAACCATCATTTCCGCCAACACAGTGGGGGTAATAGAAAAGGCCCATGAGAAAATGTTAAATCCC
GGGGCTGTGGAGGGATACATGCGCCTTCCTCTTCTGCAGAAAATCGGGCAT

>55-19_CS_151_I.69

CCCGATGGTACAACCATCATTTCCGCCAACACAGTGGGGGTAATAGAAAAGGCCCATGAGAAAATGTTAAATCCC
GGGGCTGTGGAGGGATACATGCGCCTTCCCCTTCTGCGGAAAATCGGGCAT

Section S4. HV fasta sequences.

>55-04_30317_A.01

GAGGCCGACAGCACCAGTAACAGTACTTTGTCTTCCCCGCAGAAGGGCAGCCGCACATCCGTCGAAAAAGGGACA
GCGACATCGAAAGAGGCCAAACCGAAGAAGAAGAAACGACCCCGTCTCAAA

>55-04_7352_G.01

GAGGCCGACAGCAGCAGTAACAGTACTTTGTCTTCCTCGCAGAAAAGCAGTCGGGCATCCGTCGAAAAAGGGACA
CCGACATCGAAAGAGGCCAAACCGAAGAAGAAGAAACGAGTCCATCCCAAA

>55-04_673_A.02

GAGGCCGACAGCACCAGTAACAGTACCTTGTCTTCCCCGCAGAAGGGCAGCCGCACATCCGTCGAAAAAGGGACA
GCGACATCGAAAGAGGCCAAACCGAAGAAGAAGAAACGACCCCGTCTCAAA

>55-04_369_A.03

AAGGCCGACAGCACCAGTAACAGTACTTTGTCTTCCCCGCAGAAGGGCAGCCGCACATCCGTCGAAAAAGGGACA
GCGACATCGAAAGAGGCCAAACCGAAGAAGAAGAAACGACCCCGTCTCAAA

>55-11_22273_B.01

GAGGCCGACAGCACCAGTAACAGTACTTTGTCTTCCCCGCAGAAGGGCAGCCGCACATCCGTCGAAAAAGAGACA
GCGACATCGAAAGAGGCCAAACCGAAGAAGAAGAAACGACCCCGTCTCAAA

>55-12_24827_B.01

GAGGCCGACAGCACCAGTAACAGTACTTTGTCTTCCCCGCAGAAGGGCAGCCGCACATCCGTCGAAAAAGAGACA
GCGACATCGAAAGAGGCCAAACCGAAGAAGAAGAAACGACCCCGTCTCAAA

>55-13_2407_B.01

GAGGCCGACAGCACCAGTAACAGTACTTTGTCTTCCCCGCAGAAGGGCAGCCGCACATCCGTCGAAAAAGAGACA
GCGACATCGAAAGAGGCCAAACCGAAGAAGAAGAAACGACCCCGTCTCAAA

>55-14_338_B.01

GAGGCCGACAGCACCAGTAACAGTACTTTGTCTTCCCCGCAGAAGGGCAGCCGCACATCCGTCGAAAAAGAGACA
GCGACATCGAAAGAGGCCAAACCGAAGAAGAAGAAACGACCCCGTCTCAAA

>55-15_151_B.01

GAGGCCGACAGCACCAGTAACAGTACTTTGTCTTCCCCGCAGAAGGGCAGCCGCACATCCGTCGAAAAAGAGACA
GCGACATCGAAAGAGGCCAAACCGAAGAAGAAGAAACGACCCCGTCTCAAA

>55-16_996_B.01

GAGGCCGACAGCACCAGTAACAGTACTTTGTCTTCCCCGCAGAAGGGCAGCCGCACATCCGTCGAAAAAGAGACA
GCGACATCGAAAGAGGCCAAACCGAAGAAGAAGAAACGACCCCGTCTCAAA

>55-11_1429_B.02

GAGGCCGACAGCACCAGTAACAGTACTTTGTCTTCCCCGCAGAAGGGCAGCCGCACATCCGTCGAAAAAGAGACA
GCGACATCGAAAGAGGCCAAACCGAAGAAGAAGAAACGACTCCGTCTCAAA

>55-12_1220_B.02

GAGGCCGACAGCACCAGTAACAGTACTTTGTCTTCCCCGCAGAAGGGCAGCCGCACATCCGTCGAAAAAGAGACA
GCGACATCGAAAGAGGCCAAACCGAAGAAGAAGAAACGACTCCGTCTCAAA

>55-11_189_B.04

GAGGCCGACAGCACCAGTAACAGTACTTTGTCTTCCCCGCAGAAGAGCAGCCGCACATCCGTCGAAAAAGAGACA
GCGACATCGAAAGAGGCCAAACCGAAGAAGAAGAAACGACCCCGTCTCAAA

>55-12_858_B.04

GAGGCCGACAGCACCAGTAACAGTACTTTGTCTTCCCCGCAGAAGGGCAGCCGCACATCCGTCGAAAAAGAGACA
GCGACATCGAAAGAGGCCAAACCGAAGAAGAAGAAACGACCCCATCTCAAA

>55-13_1319_B.04

GAGGCCGACAGCACCAGTAACAGTACTTTGTCTTCCCCGCAGAAGGGCAGCCGCACATCCGTCGAAAAAGAGACA
GCGACATCGAAAGAGGCCAAACCGAAGAAGAAGAAACGACCCCATCTCAAA

>55-12_222_B.05

GAGGCCGACAGCACCAGTAACAGTACTTTGTCTTCCCCGCAGAAGGGCAGCCGCACATCCGTCGAAAAGGAGACA
GCGACATCGAAAGAGGCCAAACCGAAGAAGAAGAAACGACCCCGTCTCAAA

>55-15_207_B.09

GAGGCCGACAGCACCAGTAACAGTACTTTGTCTTCCCCGCAGAAGGGCAGCCGCACATCCGTCGAAAAAGAGACA
GCGACATCGAAAGAGGCCAAACCGAAGAAGAAGAAACGACCCCGTCCCAAA

>55-16_186_B.09

GAGGCCGACAGCACCAGTAACAGTACTTTGTCTTCCCCGCAGAAGGGCAGCCGCACATCCGTCGAAAAAGAGACA
GCGACATCGAAAGAGGCCAAACCGAAGAAGAAGAAACGACCCCGTCCCAAA

>55-17_209_B.14

GAGGCCGACAGCACCAGTAACAGTACTTTGTCTTCCCCGCAGAAGGGCAGCCGCACATCCGTCGAAAAAGAGACA
GCGACATCGAAAGAGGTCAAACCGAAGAAGAAGAAACGACCCCGTCCCAAA

>55-17_227_B.12

GAGGCCGACAGCACCAGTAACAGTACTTTGTCTTCCCCGCAGAAGGGCAGCCGCACATCCGTCGAAAAAGAGACA
GCGACATCGAAAGAGGTCAAACCGAAGAAGAAGAAACGACCCCGTCTCAAA

>55-17_612_B.17

GAGGCCGACAGCACCAGTAACAGTACTTTGTCTTCCCCGCAGAAGGGCAGCCGCACATCCGTCGAAAAAGAGACA
GCGACATCGAAAGAGGTCAAACCGAAGAAGAAGAAACGACTCCGTCTCAAA

>55-11_1037_C.01

GAGGCCGACAGCACCAGTAACAGTACTTTGTCTTCCCCGCAGAAGGGCAGCCGCACATCCGTCGAAAAAGAGACA
GCGACATCGAAAGAGGCCAAACCGAAGAAGAAGAAACGAGTCCATCTCAAA

>55-13_1452_C.03

GAGGCCGACAGCACCAGTAACAGTACTTTGTCTTCCCCGCAGAAGGGCAGCCGCACATCCGTCGAAAAAGAGACA
GCGACATCGAAAGAGGCCAAACCGAAGAAGAAGAAACGAGTCCGTCTCAAA

>55-14_22205_C.03

GAGGCCGACAGCACCAGTAACAGTACTTTGTCTTCCCCGCAGAAGGGCAGCCGCACATCCGTCGAAAAAGAGACA
GCGACATCGAAAGAGGCCAAACCGAAGAAGAAGAAACGAGTCCGTCTCAAA

>55-15_10279_C.03

GAGGCCGACAGCACCAGTAACAGTACTTTGTCTTCCCCGCAGAAGGGCAGCCGCACATCCGTCGAAAAAGAGACA
GCGACATCGAAAGAGGCCAAACCGAAGAAGAAGAAACGAGTCCGTCTCAAA

>55-16_1295_C.03

GAGGCCGACAGCACCAGTAACAGTACTTTGTCTTCCCCGCAGAAGGGCAGCCGCACATCCGTCGAAAAAGAGACA
GCGACATCGAAAGAGGCCAAACCGAAGAAGAAGAAACGAGTCCGTCTCAAA

>55-17_409_C.03

GAGGCCGACAGCACCAGTAACAGTACTTTGTCTTCCCCGCAGAAGGGCAGCCGCACATCCGTCGAAAAAGAGACA
GCGACATCGAAAGAGGCCAAACCGAAGAAGAAGAAACGAGTCCGTCTCAAA

>55-14_250_C.05

GAGGCCGACAGCACCAGTAACAGTACTTTGTCTTCCCCGCAGAAGGGCAGCCACACATCCGTCGAAAAAGAGACA
GCGACATCGAAAGAGGCCAAACCGAAGAAGAAGAAACGAGTCCGTCTCAAA

>55-15_172_C.05

GAGGCCGACAGCACCAGTAACAGTACTTTGTCTTCCCCGCAGAAGGGCAGCCACACATCCGTCGAAAAAGAGACA
GCGACATCGAAAGAGGCCAAACCGAAGAAGAAGAAACGAGTCCGTCTCAAA

>55-15_159_C.06

GAGGCCGACAGCACCAGTAACAGTACTTTGTCTTCCCCGCAGAAGGGCAACCGCACATCCGTCGAAAAAGAGACA
GCGACATCGAAAGAGGCCAAACCGAAGAAGAAGAAACGAGTCCGTCTCAAA

>55-17_3038_C.09

GAGGCCGACAGCACCAGTAACAGTACTTTGTCTTCCCCGCAGAAGGGCAGCCGCACATCCGTCGAAAAAGAGACA
GCGACATCGAAAGAGGCCAAATCGAAGAAGAAGAAACGAGTCCGTCTCAAA

>55-18_6129_C.09

GAGGCCGACAGCACCAGTAACAGTACTTTGTCTTCCCCGCAGAAGGGCAGCCGCACATCCGTCGAAAAAGAGACA
GCGACATCGAAAGAGGCCAAATCGAAGAAGAAGAAACGAGTCCGTCTCAAA

>55-19_10242_C.09

GAGGCCGACAGCACCAGTAACAGTACTTTGTCTTCCCCGCAGAAGGGCAGCCGCACATCCGTCGAAAAAGAGACA
GCGACATCGAAAGAGGCCAAATCGAAGAAGAAGAAACGAGTCCGTCTCAAA

>55-17_203_C.12

GAGGCCGACAGCACCAGTAACAGTACTTTGTCTTCCCCGCAGAAGGGCAGCCGCACATCCGTCGAAAAAGAGACA
GCGACATCGAAAGAGGTCAAACCGAAGAAGAAGAAACGAGTCCGTCTCAAA

>55-12_614_C.02

GAGGCCGACAGCACCAGTAACAGTACTTTGTCTTCCCCGCAGAAGGGCAGCCGCACATCCGTCGAAAAAGAGACA
GCGACATCGAAAGAGGCCAAACCGAAGAAGAAGAAACGAGTCCGTCCCAAA

>55-13_3035_C.02

GAGGCCGACAGCACCAGTAACAGTACTTTGTCTTCCCCGCAGAAGGGCAGCCGCACATCCGTCGAAAAAGAGACA
GCGACATCGAAAGAGGCCAAACCGAAGAAGAAGAAACGAGTCCGTCCCAAA

>55-14_6038_C.02

GAGGCCGACAGCACCAGTAACAGTACTTTGTCTTCCCCGCAGAAGGGCAGCCGCACATCCGTCGAAAAAGAGACA
GCGACATCGAAAGAGGCCAAACCGAAGAAGAAGAAACGAGTCCGTCCCAAA

>55-15_3127_C.02

GAGGCCGACAGCACCAGTAACAGTACTTTGTCTTCCCCGCAGAAGGGCAGCCGCACATCCGTCGAAAAAGAGACA
GCGACATCGAAAGAGGCCAAACCGAAGAAGAAGAAACGAGTCCGTCCCAAA

>55-16_1956_C.02

GAGGCCGACAGCACCAGTAACAGTACTTTGTCTTCCCCGCAGAAGGGCAGCCGCACATCCGTCGAAAAAGAGACA
GCGACATCGAAAGAGGCCAAACCGAAGAAGAAGAAACGAGTCCGTCCCAAA

>55-16_398_C.08

GAGGCCGACAGCACCAGTAACAGTACTTTGTCTTCCCCGCAGAAGGGCGGCCGCACATCCGTCGAAAAAGAGACA
GCGACATCGAAAGAGGCCAAACCGAAGAAGAAGAAACGAGTCCGTCCCAAA

>55-17_1449_C.10

GAGGCCGACAGCACCAGTAACAGTACTTTGTCTTCCCCGCAGAAGGGCAGCCGCACATCCGTCGAAAAAGAGACA
GCGACATCGAAAGAGGCCAAACCGAAGAAGAGGAAACGAGTCCGTCTCAAA

>55-18_5251_C.10

GAGGCCGACAGCACCAGTAACAGTACTTTGTCTTCCCCGCAGAAGGGCAGCCGCACATCCGTCGAAAAAGAGACA
GCGACATCGAAAGAGGCCAAACCGAAGAAGAGGAAACGAGTCCGTCTCAAA

>55-19_6058_C.10

GAGGCCGACAGCACCAGTAACAGTACTTTGTCTTCCCCGCAGAAGGGCAGCCGCACATCCGTCGAAAAAGAGACA
GCGACATCGAAAGAGGCCAAACCGAAGAAGAGGAAACGAGTCCGTCTCAAA

>55-17_119_C.13

GAGGCCGACAGCACCAGTAACAGTACTTTGTCTTCCCCGCAGAAGGGCAGCCGCACATCCGTCGAAAAAGAGACA
GCGACATCGAAAAAGGCCAAACCGAAGAAGAGGAAACGAGTCCGTCTCAAA

>55-18_387_C.14

GAGGCCGACAGCACCAGTAACAGTACTTTGTCTTCCCCGCAGAAGGGCAGCCGCACATCCGTCGAAAAAGAGACA
GCGACATCGAAAGAGGCCAAACCGAAGAAGAGGAAACGAATCCGTCTCAAA

>55-18_232_C.15

GAGGCCGACAGCACCAGTAACAGTACTTTGTCTTCCCCGCAGAAGGGCAGCCGCACATCCGTCGAAAAAGAGACA
GCGACATCGAAAGAGGCCAAACCGAAGAAGAGGAAACAAGTCCGTCTCAAA

>55-18_130_C.16

GAGGCCGACAGCACCAGTAACAGTACTTTGTCTTCCCCGCAGAAGGGCAGCCGCACATCCGTCGAAAAAGAGACA
GCGACATCGAAAGAGGCCAAACCGAGGAAGAGGAAACGAGTCCGTCTCAAA

>55-16_709_C.07

GAGGCCGACAGCACCAGTAACAGTACTTTGTCTTCCCCGCAGAAGGGCAGCGGCACATCCGTCGAAAAAGAGACA
GCGACATCGAAAGAGGCCAAACCGAAGAAGAAGAAACGAGTCCGTCTCAAA

>55-17_152_C.11

GAGGCCGACAGCACCAGTAACAGTACTTTGTCTTCCCCGCAGAAGGGCAGCGGCACATCCGTCGAAAAAGAGACA
GCGACATCGAAAGAGGCCAAACCGAAGAAGAGGAAACGAGTCCGTCTCAAA

>55-18_227_C.11

GAGGCCGACAGCACCAGTAACAGTACTTTGTCTTCCCCGCAGAAGGGCAGCGGCACATCCGTCGAAAAAGAGACA
GCGACATCGAAAGAGGCCAAACCGAAGAAGAGGAAACGAGTCCGTCTCAAA

>55-14_446_C.04

GAGGCCGACAGCAGCAGTAACAGTACTTTGTCTTCCCCGCAGAAGGGCAGCCGCACATCCGTCGAAAAAGAGACA
GCGACATCGAAAGAGGCCAAACCGAAGAAGAAGAAACGAGTCCGTCTCAAA

>55-15_233_C.04

GAGGCCGACAGCAGCAGTAACAGTACTTTGTCTTCCCCGCAGAAGGGCAGCCGCACATCCGTCGAAAAAGAGACA
GCGACATCGAAAGAGGCCAAACCGAAGAAGAAGAAACGAGTCCGTCTCAAA

>55-19_720_C.17

GAGGCCGACAGCACCAGTAACAGTACTTTGTCTTCCCCGCAGAAGGGCAGCCGCACATCCGTCGCAAAAGAGACA
GCGACATCGAAAGAGGCCAAATCGAAGAAGAAGAAACGAGTCCGTCTCAAA

>55-11_667_B.03

GAGGCCGACAGCAGCAGTAACAGTACTTTGTCTTCCCCGCAGAAGGGCAGCCGCACATCCGTCGAAAAAGAGACA
GCGACATCGAAAGAGGCCAAACCGAAGAAGAAGAAACGACCCCGTCTCAAA

>55-12_7986_B.03

GAGGCCGACAGCAGCAGTAACAGTACTTTGTCTTCCCCGCAGAAGGGCAGCCGCACATCCGTCGAAAAAGAGACA
GCGACATCGAAAGAGGCCAAACCGAAGAAGAAGAAACGACCCCGTCTCAAA

>55-13_23031_B.03

GAGGCCGACAGCAGCAGTAACAGTACTTTGTCTTCCCCGCAGAAGGGCAGCCGCACATCCGTCGAAAAAGAGACA
GCGACATCGAAAGAGGCCAAACCGAAGAAGAAGAAACGACCCCGTCTCAAA

>55-14_3245_B.03

GAGGCCGACAGCAGCAGTAACAGTACTTTGTCTTCCCCGCAGAAGGGCAGCCGCACATCCGTCGAAAAAGAGACA
GCGACATCGAAAGAGGCCAAACCGAAGAAGAAGAAACGACCCCGTCTCAAA

>55-15_5071_B.03

GAGGCCGACAGCAGCAGTAACAGTACTTTGTCTTCCCCGCAGAAGGGCAGCCGCACATCCGTCGAAAAAGAGACA
GCGACATCGAAAGAGGCCAAACCGAAGAAGAAGAAACGACCCCGTCTCAAA

>55-16_2171_B.03

GAGGCCGACAGCAGCAGTAACAGTACTTTGTCTTCCCCGCAGAAGGGCAGCCGCACATCCGTCGAAAAAGAGACA
GCGACATCGAAAGAGGCCAAACCGAAGAAGAAGAAACGACCCCGTCTCAAA

>55-17_184_B.03

GAGGCCGACAGCAGCAGTAACAGTACTTTGTCTTCCCCGCAGAAGGGCAGCCGCACATCCGTCGAAAAAGAGACA
GCGACATCGAAAGAGGCCAAACCGAAGAAGAAGAAACGACCCCGTCTCAAA

>55-13_215_B.06

GAGGCCGACAGCAGCAGTAACAGTACTTTGTCTTCCCCGCAGAAGGGCAGCCGCACATCCGTCGAAAAGGAGAC
AGCGACATCGAAAGAGGCCAAACCGAAGAAGAAGAAACGACCCCGTCTCAAA

>55-13_194_B.07

GAGGCCGACAGCAGCAGTAACAGTACTTTGTCTTCCCCGCAGAAGGGCAGCCGCACATCCGTCGAAAAAGAGACA
GCGACATCGAAAGAGGCCAAACCGAAGAGGAAGAAACGACCCCGTCTCAAA

>55-15_213_B.08

GAGGCCGACAGCAGCAGTAACAGTACTTTGTCTTCCTCGCAGAAGGGCAGCCGCACATCCGTCGAAAAAGAGACA
GCGACATCGAAAGAGGCCAAACCGAAGAAGAAGAAACGACCCCGTCTCAAA

>55-17_217_B.13

GAGGCCGACAGCAGCAGTAACAGTACTTTGTCTTCCCCGCAGAAGGGCAGCCGCACATCCGTCGAAAAAGAGACA
GCGACATCGAAAGGGGCCAAACCGAAGAAGAAGAAACGACCCCGTCTCAAA

>55-17_180_B.16

GAGGCCGACAGCAGCAGTAACAGTACTTTGTCTTCCCCGCAGAAGGGCAGCCGCACATCCGTCGAAAAAGAGACA
GCGACATCGAAAGAGGCCAAATCGAAGAAGAAGAAACGACCCCGTCTCAAA

>55-17_994_B.11

GAGGCCGACAGCAGCAGTAACAGTACTTTGTCTTCCCCGCAGAAGGGCGGCCGCACATCCGTCGAAAAAGAGAC
AGCGACATCGAAAAAGGCCAAACCGAAGAAGAAGAAACGACCCCGTCTCAAA

>55-18_2386_B.11

GAGGCCGACAGCAGCAGTAACAGTACTTTGTCTTCCCCGCAGAAGGGCGGCCGCACATCCGTCGAAAAAGAGAC
AGCGACATCGAAAAAGGCCAAACCGAAGAAGAAGAAACGACCCCGTCTCAAA

>55-19_8983_B.11

GAGGCCGACAGCAGCAGTAACAGTACTTTGTCTTCCCCGCAGAAGGGCGGCCGCACATCCGTCGAAAAAGAGAC
AGCGACATCGAAAAAGGCCAAACCGAAGAAGAAGAAACGACCCCGTCTCAAA

>55-17_208_B.15

GAGGCCGACAGCAGCAGTAACAGTACTTTGTCTTCCCCGCAGAAGGGCAGCCGCACATCCGTCGAAAAAGAGACA
GCGACATCGAAAAAGGCCAAACCGAAGAAGAAGAAACGACCCCGTCTCAAA

>55-18_208_B.15

GAGGCCGACAGCAGCAGTAACAGTACTTTGTCTTCCCCGCAGAAGGGCAGCCGCACATCCGTCGAAAAAGAGACA
GCGACATCGAAAAAGGCCAAACCGAAGAAGAAGAAACGACCCCGTCTCAAA

>55-18_192_B.20

AAGGCCGACAGCAGCAGTAACAGTACTTTGTCTTCCCCGCAGAAGGGCGGCCGCACATCCGTCGAAAAAGAGACA
GCGACATCGAAAAAGGCCAAACCGAAGAAGAAGAAACGACCCCGTCTCAAA

>55-19_156_B.21

GAGGCCGACCGCAGCAGTAACAGTACTTTGTCTTCCCCGCAGAAGGGCGGCCGCACATCCGTCGAAAAAGAGACA
GCGACATCGAAAAAGGCCAAACCGAAGAAGAAGAAACGACCCCGTCTCAAA

>55-18_194_B.18

AAGGCCGACAGCAGCAGTAACAGTACTTTGTCTTCCCCGCAGAAGGGCAGCCGCACATCCGTCGAAAAAGAGACA
GCGACATCGAAAGAGGCCAAACCGAGGAAGAAGAAACGACCCCGTCTCAAA

>55-19_769_B.18

AAGGCCGACAGCAGCAGTAACAGTACTTTGTCTTCCCCGCAGAAGGGCAGCCGCACATCCGTCGAAAAAGAGACA
GCGACATCGAAAGAGGCCAAACCGAGGAAGAAGAAACGACCCCGTCTCAAA

>55-15_152_B.10

GAGGCCGACAGCAGCCGTAACAGTACTTTGTCTTCCCCGCAGAAGGGCAGCCGCACATCCGTCGAAAAAGAGACA
GCGACATCGAAAGAGGCCAAACCGAAGAAGAAGAAACGACCCCGTCTCAAA

>55-18_545_B.19

GAGGCCGACAGCAGCAGTAACAGTACTTTGTCTTCCCCGCAGAAGGGCAGCCGCACATCCGTCGAAAAAGAGACA
GCGACATCGAAAGAGGGCAAACCGAAGAAGAAGAAACGACCCCGTCTCAAA

>55-11_528_D.01

GAGGCCGACAGCACCAGTAACAGTACTTTGTCTTCCCCGCAGAAGGGCAGCCGCGCATCCGTCGAAAGAGGGAC
AGCGACATCGAAAGAGGCCAAACCGAAGAAGAAGAAACGACTCCGTCTCAAA

>55-14_337_D.01

GAGGCCGACAGCACCAGTAACAGTACTTTGTCTTCCCCGCAGAAGGGCAGCCGCGCATCCGTCGAAAGAGGGAC
AGCGACATCGAAAGAGGCCAAACCGAAGAAGAAGAAACGACTCCGTCTCAAA

>55-15_403_D.01

GAGGCCGACAGCACCAGTAACAGTACTTTGTCTTCCCCGCAGAAGGGCAGCCGCGCATCCGTCGAAAGAGGGAC
AGCGACATCGAAAGAGGCCAAACCGAAGAAGAAGAAACGACTCCGTCTCAAA

>55-16_4768_D.01

GAGGCCGACAGCACCAGTAACAGTACTTTGTCTTCCCCGCAGAAGGGCAGCCGCGCATCCGTCGAAAGAGGGAC
AGCGACATCGAAAGAGGCCAAACCGAAGAAGAAGAAACGACTCCGTCTCAAA

>55-17_205_D.01

GAGGCCGACAGCACCAGTAACAGTACTTTGTCTTCCCCGCAGAAGGGCAGCCGCGCATCCGTCGAAAGAGGGAC
AGCGACATCGAAAGAGGCCAAACCGAAGAAGAAGAAACGACTCCGTCTCAAA

>55-16_386_D.02

GAGGCCGACAGCACCAGTAACAGTACTTTGTCTTCCCCGCAGAAGGGCAGCCGCGCATCCGTCGAAAGAGGGAC
AGCGACATCGAAAGAGGCCAAATCGAAGAAGAAGAAACGACTCCGTCTCAAA

>55-17_545_D.02

GAGGCCGACAGCACCAGTAACAGTACTTTGTCTTCCCCGCAGAAGGGCAGCCGCGCATCCGTCGAAAGAGGGAC
AGCGACATCGAAAGAGGCCAAATCGAAGAAGAAGAAACGACTCCGTCTCAAA

>55-16_445_D.03

GAGGCCGACAGCACCAGTAACAGTACTTTGTCTTCCCCGCAGAAGGGCAGCCGCGCATCCGTCGAAAGAGGGAC
AGCGACATCGAAAGAGGCCAAACCGAAGAAGAAGAAACGACTCCGTCCCAAA

>55-16_323_D.04

GAGGCCGACAGCACCAGTAACAGTACTTTGTCTTCCCCGCAGAAGGGCAGCCGCGCATCCGTCGAAAGAGGGAC
AGCGACATCGAAAGAGGCCAAACCGAAGAAGAAGAAACGACCCCGTCTCAAA

>55-17_1604_D.05

GAGGCCGACAGCACCAGTAACAGTACTTTGTCTTCCCCGCAGAAGGGCAGCCGCGCATCCGTCGAAAGAGGGAC
AGCGACATCGAAAGAGGCCAAACCGAAGAAGAGGAAACGACTCCGTCTCAAA

>55-18_426_D.05

GAGGCCGACAGCACCAGTAACAGTACTTTGTCTTCCCCGCAGAAGGGCAGCCGCGCATCCGTCGAAAGAGGGAC
AGCGACATCGAAAGAGGCCAAACCGAAGAAGAGGAAACGACTCCGTCTCAAA

>55-19_1227_D.05

GAGGCCGACAGCACCAGTAACAGTACTTTGTCTTCCCCGCAGAAGGGCAGCCGCGCATCCGTCGAAAGAGGGAC
AGCGACATCGAAAGAGGCCAAACCGAAGAAGAGGAAACGACTCCGTCTCAAA

>55-17_612_D.07

GAGGCCGACAGCACCAGTAACAGTACTTTGTCTTCCCCGCAGAAGGGCAGCCGCGCATCCGTCGAAAGAGGGAC
AGCGACATCGAAAGAGGTCAAACCGAAGAAGAGGAAACGACTCCGTCTCAAA

>55-18_175_D.07

GAGGCCGACAGCACCAGTAACAGTACTTTGTCTTCCCCGCAGAAGGGCAGCCGCGCATCCGTCGAAAGAGGGAC
AGCGACATCGAAAGAGGTCAAACCGAAGAAGAGGAAACGACTCCGTCTCAAA

>55-17_624_D.06

GAGGCCGACAGCACCAGTAACAGTACTTTGTCTTCCCCGCAGAAGGGCAGCCGCGCATCCGTCGAAAGAGGGAC
AGCGACATCGAAAGAGGTCAAACCGAAGAAGAAGAAACGACTCCGTCTCAAA

>55-17_143_D.09

GAGGCCGACAGCACCAGTAACAGTACTTTGTCTTCCCCGCAGAAGGGCAGCCGCGCATCCGTCGAAAGAGGGAC
AGCGACATCGAAAGAGGGCAAACCGAAGAAGAAGAAACGACTCCGTCTCAAA

>55-18_166_D.10

GAGGCCGACAGCACCAGTAACAGTACTTTGTCTTCCCCGCAGAAGGGCAGCCGCGCATCCGTCGAAAGAGGGAC
AGCGACATCGAAAGAGCCCAAACCGAAGAAGAAGAAACGACTCCGTCTCAAA

>55-19_391_D.06

GAGGCCGACAGCACCAGTAACAGTACTTTGTCTTCCCCGCAGAAGGGCAGCCGCGCATCCGTCGAAAGAGGGAC
AGCGACATCGAAAGAGGCCAAACCGACGAAGAAGAAACGACTCCGTCTCAAA

>55-17_167_D.08

GAGGCCGACAGCACCAGTAACAGTACTTTGTCTTCCCCGCAGAAGGGCAGCCGCGCATCCGTCGAAAGAGGGAG
AGCGACATCGAAAGAGGCCAAACCGAAGAAGAGGAAACGACTCCGTCTCAAA

>55-11_621_A.05

GAGGCCGACAGCACCAGTAACAGTACTTTGTCTTCCCCGCAGAAGGGCAGCCGCACATCCGTCGAAAAAGGGAAA
GCGACATCGAAAGAGGCCAAACCGAAGAAGAAGAAACGACTCCGTCTCAAA

>55-12_248_A.05

GAGGCCGACAGCACCAGTAACAGTACTTTGTCTTCCCCGCAGAAGGGCAGCCGCACATCCGTCGAAAAAGGGAAA
GCGACATCGAAAGAGGCCAAACCGAAGAAGAAGAAACGACTCCGTCTCAAA

>55-14_161_A.05

GAGGCCGACAGCACCAGTAACAGTACTTTGTCTTCCCCGCAGAAGGGCAGCCGCACATCCGTCGAAAAAGGGAAA
GCGACATCGAAAGAGGCCAAACCGAAGAAGAAGAAACGACTCCGTCTCAAA

>55-11_651_A.04

GAGGCCGACAGCACCAGTAACAGTACTTTGTCTTCCCCGCAGAAGGGCCGCCGCACATCCGTCGAAAAAGGGACA GCGACATCGAAAGAGGCCAAACCGAAGAAGAAGAAACGACTCCGTCTCAAA

>55-11_2013_E.01

GAGGCCGACAGCACCAGTAACAGTACTTTGTCTTCCCCGCAGAAGGGCAGCCGCACATCCGTCGAAAAAGGGAG AGCGACATCGAAAGAGGCCAAACCGAAGAAGAAGAAACGAGTCCATCTCAAA

>55-11_200_E.02

GAGGCCGACAGCACCAGTAACAGTACTTTGTCTTCCCCGCAGAAGGGCAGCCGCACATTCGTCAAAAAAGGGACA GCGACATCGAAAGAGGCCAAACCGAAGAAGAAGAAACGAGTCCATCTCAAA

>55-14_550_F.01

GAGGCCGACAGCACCAGTAACAGTACTTTGTCTTCCCCGCAGAAGGGCAGCCGCGCATCCGTCGAAAAAGGGATA GCGACATCGAAAGAGGCCAAACCGAAGAAGAAGAAACGAGTCCGTCCCAAA

>55-16_352_F.01

GAGGCCGACAGCACCAGTAACAGTACTTTGTCTTCCCCGCAGAAGGGCAGCCGCGCATCCGTCGAAAAAGGGATA GCGACATCGAAAGAGGCCAAACCGAAGAAGAAGAAACGAGTCCGTCCCAAA

>55-17_119_F.01

GAGGCCGACAGCACCAGTAACAGTACTTTGTCTTCCCCGCAGAAGGGCAGCCGCGCATCCGTCGAAAAAGGGATA GCGACATCGAAAGAGGCCAAACCGAAGAAGAAGAAACGAGTCCGTCCCAAA

>55-17_6406_F.03

GAGGCCGACAGCACCAGTAACAGTACTTTGTCTTCCCCGCAGAAGGGCAGCCGCGCATCCGTCGAAAAAGGGATA GCGACATCGAAAGAGGCCAAACCGAAGAAGAAGAAAGGAGTCCGTCCCAAA

>55-18_2445_F.03

GAGGCCGACAGCACCAGTAACAGTACTTTGTCTTCCCCGCAGAAGGGCAGCCGCGCATCCGTCGAAAAAGGGATA GCGACATCGAAAGAGGCCAAACCGAAGAAGAAGAAAGGAGTCCGTCCCAAA

>55-19_1008_F.03

GAGGCCGACAGCACCAGTAACAGTACTTTGTCTTCCCCGCAGAAGGGCAGCCGCGCATCCGTCGAAAAAGGGATA GCGACATCGAAAGAGGCCAAACCGAAGAAGAAGAAAGGAGTCCGTCCCAAA

>55-18_136_F.04

GAGGCCGACAACACCAGTAACAGTACTTTGTCTTCCCCGCAGAAGGGCAGCCGCGCATCCGTCAAAAAAGGGATA GCGACATCGAAAGAGGCCAAACCGAAGAAGAAGAAAGGAGTCCGTCCCAAA

>55-17_9306_F.02

GAGGCCGACAGCACCAGTAACAGTACTTTGTCTTCCCCGCAGAAGGGCAGCCGCGCATCCGTCGAAAAAGGGATA GCGACATCGAAAGAGGGCAAACCGAAGAAGAAGAAAGGAGTCCGTCCCAAA

>55-18_9241_F.02

GAGGCCGACAGCACCAGTAACAGTACTTTGTCTTCCCCGCAGAAGGGCAGCCGCGCATCCGTCGAAAAAGGGATA
GCGACATCGAAAGAGGGCAAACCGAAGAAGAAGAAAGGAGTCCGTCCCAAA

>55-19_7876_F.02

GAGGCCGACAGCACCAGTAACAGTACTTTGTCTTCCCCGCAGAAGGGCAGCCGCGCATCCGTCGAAAAAGGGATA
GCGACATCGAAAGAGGGCAAACCGAAGAAGAAGAAAGGAGTCCGTCCCAAA

>55-15_516_E.03

GAGGCCGACAGCACCAGTAACAGTACTTTGTCTTCCCCGCAGAAGGGCATCCGCACATCCGTCGAAAAAGGGATA
GCGACATCGAAAGAGGCCAAACCGAAGAAGAAGAAACGAGTCCATCTCAAA

>55-16_1725_E.03

GAGGCCGACAGCACCAGTAACAGTACTTTGTCTTCCCCGCAGAAGGGCATCCGCACATCCGTCGAAAAAGGGATA
GCGACATCGAAAGAGGCCAAACCGAAGAAGAAGAAACGAGTCCATCTCAAA

>55-17_247_E.03

GAGGCCGACAGCACCAGTAACAGTACTTTGTCTTCCCCGCAGAAGGGCATCCGCACATCCGTCGAAAAAGGGATA
GCGACATCGAAAGAGGCCAAACCGAAGAAGAAGAAACGAGTCCATCTCAAA

>55-18_331_E.03

GAGGCCGACAGCACCAGTAACAGTACTTTGTCTTCCCCGCAGAAGGGCATCCGCACATCCGTCGAAAAAGGGATA
GCGACATCGAAAGAGGCCAAACCGAAGAAGAAGAAACGAGTCCATCTCAAA

>55-18_151_E.05

GAGGCCGACAGCACCAGTAACAGTACTTTGTCTTCCCCGCAGAAGGGCATCCGCACATCCGTCGAAAAAGGGATA
ACGACATCGAAAGAGGCCAAACCGAAGAAGAAGAAACGAGTCCATCTCAAA

>55-16_555_E.04

GAGGCCGACAGCACCAGTAACAGTACTTTGTCTTCCCCGCAGAAGGGCATCCGCACATCCGTCGAAAAAGGGATA
GCGACATCGAAAGAGGCCAAACCGAAGAAGAAGAAACGAGTCCGTCCCAAA

>55-19_385_E.06

GAGGCCGACAGCACCAGTAACAGTACTTTGTCTTCCCCGCAGAAGGACATCCGCACATCCGTCGAAAAAGGGATA
GCGACATCGAAAGAGGCCAAACCGAAGAAGAGGAAACGAGTCCATCTCAAA

>55-04_4830_G.02

GAGGCCGACAGCAGCAGTAACAGTACTTTGTCTTCCCCGCAGAAAAGCAGTCGGGCATCCGTCGAAAAAGGGAC
ACCGACATCGAAAGAGGCCAAACCGAAGAAGAAGAAACGAGTCCATCCCAAA

>55-11_221_G.02

GAGGCCGACAGCAGCAGTAACAGTACTTTGTCTTCCCCGCAGAAAAGCAGTCGGGCATCCGTCGAAAAAGGGAC
ACCGACATCGAAAGAGGCCAAACCGAAGAAGAAGAAACGAGTCCATCCCAAA

>55-11_14151_G.03

GAGGCCGACAGCAGCAGTAACAGTACTTTGTCTTCCCCGCAGAAAAGCAGTCGGGCATCCGTCGAAAAAGGGAC
ACCGACATCGAAAGAGGCCAAACCGAAGAAGAAGAAACGAGTCCGTCCCAAA

>55-12_18736_G.03

GAGGCCGACAGCAGCAGTAACAGTACTTTGTCTTCCCCGCAGAAAAGCAGTCGGGCATCCGTCGAAAAAGGGAC
ACCGACATCGAAAGAGGCCAAACCGAAGAAGAAGAAACGAGTCCGTCCCAAA

>55-13_3137_G.03

GAGGCCGACAGCAGCAGTAACAGTACTTTGTCTTCCCCGCAGAAAAGCAGTCGGGCATCCGTCGAAAAAGGGAC
ACCGACATCGAAAGAGGCCAAACCGAAGAAGAAGAAACGAGTCCGTCCCAAA

>55-14_3777_G.03

GAGGCCGACAGCAGCAGTAACAGTACTTTGTCTTCCCCGCAGAAAAGCAGTCGGGCATCCGTCGAAAAAGGGAC
ACCGACATCGAAAGAGGCCAAACCGAAGAAGAAGAAACGAGTCCGTCCCAAA

>55-15_9194_G.03

GAGGCCGACAGCAGCAGTAACAGTACTTTGTCTTCCCCGCAGAAAAGCAGTCGGGCATCCGTCGAAAAAGGGAC
ACCGACATCGAAAGAGGCCAAACCGAAGAAGAAGAAACGAGTCCGTCCCAAA

>55-16_6108_G.03

GAGGCCGACAGCAGCAGTAACAGTACTTTGTCTTCCCCGCAGAAAAGCAGTCGGGCATCCGTCGAAAAAGGGAC
ACCGACATCGAAAGAGGCCAAACCGAAGAAGAAGAAACGAGTCCGTCCCAAA

>55-18_217_G.03

GAGGCCGACAGCAGCAGTAACAGTACTTTGTCTTCCCCGCAGAAAAGCAGTCGGGCATCCGTCGAAAAAGGGAC
ACCGACATCGAAAGAGGCCAAACCGAAGAAGAAGAAACGAGTCCGTCCCAAA

>55-11_352_G.05

GAGGCCGACAGCAGCAGTAACAGTACTTTGTCTTCCCCGCAGAAAAGCAGTCGGGCATCCGTCGAAAAAGGGAC
ACCGACATCGAAAGAGGCCAAACCGAAGAAGAAGAAACGAGTCCGTCTCAAA

>55-16_284_G.05

GAGGCCGACAGCAGCAGTAACAGTACTTTGTCTTCCCCGCAGAAAAGCAGTCGGGCATCCGTCGAAAAAGGGAC
ACCGACATCGAAAGAGGCCAAACCGAAGAAGAAGAAACGAGTCCGTCTCAAA

>55-11_204_G.06

GAGGCCGACAGCAGCAGTAACAGTACTTTGTCTTCCCCGCAGAAAAGCAGTCGGGCATCCGTCGAAAAAGGGAC
ACCAACATCGAAAGAGGCCAAACCGAAGAAGAAGAAACGAGTCCGTCCCAAA

>55-12_211_G.07

GAGGCCGACAGCAGCAGTAACAGTACTTTGTCTTCCCCGCAGAAAAGCGGTCGGGCATCCGTCGAAAAAGGGAC
ACCGACATCGAAAGAGGCCAAACCGAAGAAGAAGAAACGAGTCCGTCCCAAA

>55-14_1053_G.08

GAGGCCGACAGCAGCAGTAACAGTACTTTGTCTTCCCCGCAGAAAAGCAGTCGGGCATCCGTCGAAAAAGGGAC
ACCGACATCGAAAGAGGCCAAATCGAAGAAGAAGAAACGAGTCCGTCCCAAA

>55-15_3873_G.08

GAGGCCGACAGCAGCAGTAACAGTACTTTGTCTTCCCCGCAGAAAAGCAGTCGGGCATCCGTCGAAAAAGGGAC
ACCGACATCGAAAGAGGCCAAATCGAAGAAGAAGAAACGAGTCCGTCCCAAA

>55-16_4320_G.08

GAGGCCGACAGCAGCAGTAACAGTACTTTGTCTTCCCCGCAGAAAAGCAGTCGGGCATCCGTCGAAAAAGGGAC
ACCGACATCGAAAGAGGCCAAATCGAAGAAGAAGAAACGAGTCCGTCCCAAA

>55-17_1026_G.08

GAGGCCGACAGCAGCAGTAACAGTACTTTGTCTTCCCCGCAGAAAAGCAGTCGGGCATCCGTCGAAAAAGGGAC
ACCGACATCGAAAGAGGCCAAATCGAAGAAGAAGAAACGAGTCCGTCCCAAA

>55-18_1020_G.08

GAGGCCGACAGCAGCAGTAACAGTACTTTGTCTTCCCCGCAGAAAAGCAGTCGGGCATCCGTCGAAAAAGGGAC
ACCGACATCGAAAGAGGCCAAATCGAAGAAGAAGAAACGAGTCCGTCCCAAA

>55-19_640_G.08

GAGGCCGACAGCAGCAGTAACAGTACTTTGTCTTCCCCGCAGAAAAGCAGTCGGGCATCCGTCGAAAAAGGGAC
ACCGACATCGAAAGAGGCCAAATCGAAGAAGAAGAAACGAGTCCGTCCCAAA

>55-15_216_G.11

GAGGCCGACAGCAGTAGTAACAGTACTTTGTCTTCCCCGCAGAAAAGCAGTCGGGCATCCGTCGAAAAAGGGACA
CCGACATCGAAAGAGGCCAAACCGAAGAAGAAGAAACGAGTCCGTCCCAAA

>55-11_527_G.04

GAGGCCGACAGCACCAGTAACAGTACTTTGTCTTCCCCGCAGAAAAGCAGTCGGGCATCCGTCGAAAAAGGGACA
CCGACATCGAAAGAGGCCAAACCGAAGAAGAAGAAACGAGTCCGTCCCAAA

>55-14_209_G.04

GAGGCCGACAGCACCAGTAACAGTACTTTGTCTTCCCCGCAGAAAAGCAGTCGGGCATCCGTCGAAAAAGGGACA
CCGACATCGAAAGAGGCCAAACCGAAGAAGAAGAAACGAGTCCGTCCCAAA

>55-14_341_G.09

GAGGCCGACAGCAGCAGTAACAGTACTTTGTCTTCCCCGCAGAAAAGCAGTCGCGCATCCGTCGAAAAAGGGACA
CCGACATCGAAAGAGGCCAAACCGAAGAAGAAGAAACGAGTCCGTCCCAAA

>55-15_3011_G.09

GAGGCCGACAGCAGCAGTAACAGTACTTTGTCTTCCCCGCAGAAAAGCAGTCGCGCATCCGTCGAAAAAGGGACA
CCGACATCGAAAGAGGCCAAACCGAAGAAGAAGAAACGAGTCCGTCCCAAA

>55-16_752_G.09

GAGGCCGACAGCAGCAGTAACAGTACTTTGTCTTCCCCGCAGAAAAGCAGTCGCGCATCCGTCGAAAAAGGGACA CCGACATCGAAAGAGGCCAAACCGAAGAAGAAGAAACGAGTCCGTCCCAAA

>55-19_1550_G.09

GAGGCCGACAGCAGCAGTAACAGTACTTTGTCTTCCCCGCAGAAAAGCAGTCGCGCATCCGTCGAAAAAGGGACA CCGACATCGAAAGAGGCCAAACCGAAGAAGAAGAAACGAGTCCGTCCCAAA

>55-15_219_G.10

GAGGCCGACAGCAGCAGTAACAGTACTTTGTCTTCCCCGCAGAAAAGCAGTCGCGCATCCGTCGAAAAAGGGACA CCGACATCGAAAGAGGCCAAACCGAAGAAGAAGAAACGAATCCGTCCCAAA

>55-19_362_G.10

GAGGCCGATAGCAGCAGTAACAGTACTTTGTCTTCCCCGCAGAAAAGCAGTCGCGCATCCGTCGAAAAAGGGACA CCGACATCGAAAGAGGCCAAACCGAAGAAGAAGAAACGAGTCCGTCCCAAA

>55-11_347_H.01

GAGGCCGACAGCAGCAGTAACAGTACTTTGTCTTCCCCGCAGAAAAGCAGTCGGGCATCCGTCGAAAAAGAGACA GCGACATCGAAAGAGGCCAAACCGAAGAAGAAGAAACGACCCCGTCTCAAA

>55-11_247_H.02

GAGGCCGACAGCAGCAGTAACAGTACTTTGTCTTCCCCGCAGAAAAGCAGTCGGGCATCCGTCGAAAAAGGGAC ACCGACATCGAAAGAGGCCAAACCGAAGAAGAAGAAACGACCCCGTCTCAAA
